# Author Correction: CD98hc is a target for brain delivery of biotherapeutics

**DOI:** 10.1038/s41467-023-41355-x

**Published:** 2023-09-07

**Authors:** Kylie S. Chew, Robert C. Wells, Arash Moshkforoush, Darren Chan, Kendra J. Lechtenberg, Hai L. Tran, Johann Chow, Do Jin Kim, Yaneth Robles-Colmenares, Devendra B. Srivastava, Raymond K. Tong, Mabel Tong, Kaitlin Xa, Alexander Yang, Yinhan Zhou, Padma Akkapeddi, Lakshman Annamalai, Kaja Bajc, Marie Blanchette, Gerald Maxwell Cherf, Timothy K. Earr, Audrey Gill, David Huynh, David Joy, Kristen N. Knight, Diana Lac, Amy Wing-Sze Leung, Katrina W. Lexa, Nicholas P. D. Liau, Isabel Becerra, Mario Malfavon, Joseph McInnes, Hoang N. Nguyen, Edwin I. Lozano, Michelle E. Pizzo, Elysia Roche, Patricia Sacayon, Meredith E. K. Calvert, Richard Daneman, Mark S. Dennis, Joseph Duque, Kapil Gadkar, Joseph W. Lewcock, Cathal S. Mahon, René Meisner, Hilda Solanoy, Robert G. Thorne, Ryan J. Watts, Y. Joy Yu Zuchero, Mihalis S. Kariolis

**Affiliations:** 1 Denali Therapeutics, Inc., 161 Oyster Point Blvd., South San Francisco, CA 94080 USA; 2 Department of Pharmacology, University of California San Diego, 9500 Gilman Dr., La Jolla, CA 92093 USA; 3 Department of Neurosciences, University of California San Diego, 9500 Gilman Dr., La Jolla, CA 92093 USA; 4 Department of Pharmaceutics, University of Minnesota, Minneapolis, MN USA

**Keywords:** Blood-brain barrier, Drug delivery, Protein design, X-ray crystallography, Computational models

Correction to: *Nature Communications* 10.1038/s41467-023-40681-4, published online 19 August 2023

The original version of this Article contained errors in Supplementary Fig. 7 panels a, b, c, d, e, g, and h, in which the units on the *x* axis incorrectly read ‘Time (hrs)’ rather than the correct ‘Time (day)’. The HTML has been updated to include a corrected version of the Supplementary Information.

### Supplementary information

Updated Supplementary Information

