# Supplementary material for: CD98hc is a target for brain delivery of biotherapeutics
Source: Nat Commun. 2023 Aug 19;14:5053. doi: 10.1038/s41467-023-40681-4 (PMC10439950; doi:10.1038/s41467-023-40681-4)
Supplement: Supplementary file 1 — Supplementary Information [file 41467_2023_40681_MOESM1_ESM.pdf]

CD98hc is a target for brain delivery of biotherapeutics

Authors: Kylie S. Chew[1] *, Robert C. Wells[1] *, Arash Moshkforoush[1], Darren Chan[1], Kendra J. Lechtenberg[1], Hai L. Tran[1], Johann Chow[1], Do Jin Kim[1], Yaneth Robles-Colmenares[1], Devendra B. Srivastava[1], Raymond K. Tong[1], Mabel Tong[1], Kaitlin Xa[1], Alexander Yang[1], Yinhan Zhou[1], Padma Akkapeddi[1], Lakshman Annamalai[1], Kaja Bajc[2,3], Marie Blanchette[2,3], Gerald Maxwell Cherf [1], Timothy K. Earr[1], Audrey Gill[1], David Huynh[1], David Joy[1], Kristen N. Knight[1], Diana Lac[1], Amy Wing-Sze Leung[1], Katrina W. Lexa[1], Nicholas P. D. Liau[1], Isabel Becerra[1], Mario Malfavon[2,3], Joseph McInnes[1], Hoang N. Nguyen[1], Edwin I. Lozano[1], Michelle E. Pizzo[1], Elysia Roche[1], Patricia Sacayon[1], Meredith E. K. Calvert [1], Richard Daneman[2,3], Mark S. Dennis[1], Joseph Duque[1], Kapil Gadkar[1], Joseph W. Lewcock[1], Cathal S. Mahon[1], René Meisner[1], Hilda Solanoy[1], Robert G. Thorne[1,4], Ryan J. Watts[1], Y. Joy Yu Zuchero[1] ^, and Mihalis S Kariolis[1] ^

* These authors contributed equally: Kylie S. Chew, Robert C. Wells
^ email: zuchero@dnli.com; kariolis@dnli.com

Affiliations: [1]Denali Therapeutics, Inc., 161 Oyster Point Blvd., South San Francisco, CA 94080

[2]Department of Pharmacology, University of California San Diego, 9500 Gilman Dr., La Jolla, CA, USA. 92093

[3]Department of Neurosciences, University of California San Diego, 9500 Gilman Dr., La Jolla, CA, USA. 92093

[4]Department of Pharmaceutics, University of Minnesota, Minneapolis, MN

Supplemental materials and methods

TV library designs

Design of the limited liability β-sheet library

Design of the TV β-sheet library was done off a structural assessment of the human Fc domain (PDB 4W4O), resulting in a set of positions selected for diversification; human Fc residues 380, 382, 384-387, 422, 424, 426, 438 and 440 (EU numbering). Diversification was performed using a "limited liability" approach designed to reduce the number, and relative position, of amino acids associated with developability risks. These residues include arginine and tryptophan, which are known to enhance the affinity of an interaction, but also enhance non-specificity, cysteine, which may cause aberrant disulfides, and glycine, which increases the flexibility of protein structure and can lead destabilization of secondary structure. A combination of NNK and NHK codons were used to achieve the limited liability bias. NNK encodes for all 20 amino acids, while NHK encodes for all amino acids, except for arginine, tryptophan, glycine, and cysteine. NNK and NHK codons were alternated during library synthesis to reduce the overall number of amino acids associated with liabilities, as well as to prevent them from occupying neighboring positions.

To generate the "limited liability" β-sheet library, six oligonucleotide fragments containing NNK and NHK diversification at specific positions (**Supplemental Figure 1a**) were pooled and constructed using Kunkle mutagenesis to create nine unique TV β-sheet libraries using all combinations of these six fragments. The library was transformed into TG1 cells and infected with CM13 helper phage to generate phage displaying a library of TV fragments. The phage library was precipitated and used to pan against huCD98hc.

Design of the affinity maturation libraries of TV6 (AM1-AM4)

Using TV6 as the template, affinity maturation libraries 1 and 2 (AM1, AM2) were designed to optimize the composition of the original library positions as outlined in **Supplemental Figure 2a**. A combination of NNK codons and restricted degenerate codons were used to incorporate diversity at specified positions. Restricted diversity was used to bias the libraries towards amino acids found to be beneficial in the initial set of hits. Libraries were made using Kunkel mutagenesis, cloned into the phage display vector, and panned against huCD98hc.

Using TV6.1 as a template, affinity maturation libraries 3 and 4 (AM3 and AM4) were designed as outlined in **Supplemental Figure 2b**, with the aim of interrogating an expanded

sequence space outside of original library positions. Diversification was done using NHK codons and the limited liability approach, with several positions further restricted to amino acids known to be favorable for binding (positions 386 and 441 in AM3 and AM4, respectively). Libraries were constructed using Kunkel mutagenesis, cloned into the phage display vector, and panned against huCD98hc.

Design of the affinity maturation patch libraries of TV6.8

A set of ten patch libraries were designed by varying 4 or 5 positions within, or proximal to, the TV patch (**Supplemental Figure 2c**). TV6.8 was used as a template and selected positions were randomized using NNK codons. Libraries were built by PCR and transformed into yeast for display on the yeast cell surface and sorted to isolate variants with improved binding as described above.

Design of the TV6 family dematuration clones

Clones with weaker binding to huCD98hc were rationally designed by reverting one or more residues in TV6.8 to the wild-type amino acid or to residues shown previously to moderately affect the affinity to the human CD98hc ECD.

CD98hc$^{mu/hu}$ KI mice generation and characterization

Construct design and mouse generation

The construct for humanizing the extracellular domain (ECD) of CD98hc contained 5 primary elements **(Supplemental Figure 5a)**. First, 3' and 5' arms homologous to the endogenous mouse SLC3A2 locus to enable homologous recombination. Next, point mutations were made in murine exons 2, 3, and 4 to humanize only those extracellular mouse residues that differ from the orthologous human residues. This second element enabled preservation of mouse introns 1, 2, and 3, which are predicted to have promoter regulatory regions, and the endogenous splice sites at the intron1-exon1/2, intron2-exon 2/3, intron3-exon 3/4, and intron4-exon4 junctions. The third element was an FRT flanked Neo cassette (neomycin resistance gene) into murine intron 4, which served to disrupt a long region of mouse homology that could have caused incomplete incorporation of the entire construct and enabled screening for partial incorporation based on neomycin antibiotic resistance. Because intron 4 also contained predicted promoter regulatory

regions we were concerned the Neo cassette could disrupt *Slc3a2* expression therefore the FRT sites provided the option to remove the cassette in ES after incorporation was confirmed. The fourth element was the cDNA of human residues E335 to the STOP codon in place of the murine genomic DNA from residue S268 in exon 5 to the STOP codon in exon 10, which achieved humanization of the remainder of the CD98hc ECD while providing enough differentiation from the endogenous mouse sequence that homologous recombination of the entire construct could be achieved. The fifth element was a F3' flanked hygro cassette (hygromycin resistance gene) downstream of the murine 3' UTR, which enabled screening for incorporation of the entire construct by adding hygromycin in addition to neomycin to the ES cell culture medium. Because the hygromycin was after the stop codon, we did not anticipate it disrupting Slc3a2 expression and the cassette would be automatically excised in the male germline of the resulting mice. **Supplemental Figure 5a** illustrates the construct designed for knocking-in a humanized ECD CD98hc mouse. This construct was electroporated into ES cells from C57Bl6 mice. ES cells with proper homologous recombination were selected for by growing the cells in the presence of neomycin and hygromycin. Incorporation was confirmed by PCR. The Neo cassette was removed *in vitro* by electroporation of a Flp recombinase expressing construct. This step was critical as the Neo cassette was suspected to disrupt expression of the SLC3A2 gene, and CD98hc protein is required for sperm function. ES cells containing the properly incorporated humanized SLC3A2 gene without the Neo cassette were injected into goGermline blastocytes (Ozgene)(*1*), followed by embryo transfer to pseudo pregnant females. Founder males were selected from the offspring of the female that received the embryos and bred to wild-type females to generate F1 heterozygous mice. Homozygous mice were subsequently generated from breeding of F1 generation heterozygous mice.

CD98hc$^{mu/hu}$ KI mice characterization

Wild-type (C57/B6) control, CD98hc$^{mu/hu}$ KI/WT, and CD98hc$^{mu/hu}$ KI/KI animals were sacrificed and perfused with PBS, and hemi-brains, kidney, testis, large intestine, and pancreas were drop fixed in 4% PFA overnight followed by 30% sucrose preservation. Additionally, pieces of brain, small intestine, kidney, lung, and liver were snap frozen.

For fixed tissues, 10 μm sections (sagittal for brain) were cut using a cryostat and mounted on superfrost slides, blocked in 5% BSA + 0.3% Triton X-100, followed by staining for mouse or

human CD98hc (**Supplemental Table 8**). Brain sections were also co-stained for Glut1 to identify the brain vasculature. Images of brain sections were taken using a Leica SP8 Lightning confocal microscope with a 20x objective running Leica Application Suite X (3.5.7.23225) (**Supplemental Figure 5b**). Peripheral tissue images were taken with a Zeiss Axio Scan with a 20x objective running ZEN3.5 (Zeiss) (**Supplemental Figure 5c-h)**

Frozen tissues were homogenized using a Qiagen TissueLyser in 10x tissue weight of lysis buffer containing 1% NP-40 + protease inhibitors in PBS. Total protein concentration was measured with BCA and samples were diluted to 1 mg/mL of total protein in NuPAGE LDS sample buffer (4X) with NuPAGE sample reducing agent (10X) and boiled for 10 minutes. Samples were run on NuPAGE 4-12% Bis-Tris gels. Because anti-CD98hc antibodies are either mouse or human CD98hc specific, protein samples were run with dilutions (50, 10, 2, 0.4, 0.08 µg/mL) of either mouse or human CD98hc recombinant protein on the same gels. Mouse CD98hc was detected with rabbit anti-mouse CD98hc and human CD98hc was detected with rabbit anti-human CD98hc. Blots were cut and co-stained with rabbit anti-GAPDH as a loading control. Primary antibodies were detected with licor secondaries. See **Supplemental Table 7** for details on primary and secondary antibodies. Blots were imaged on a Li-Cor Odyssey CLx (**Supplemental Figure 5i**) and quantified with Image Studio Lite (Li-Cor, Version 5.2.5). Concentrations of CD98hc were calculated based on a standard curve of recombinant proteins (**Supplemental Figure 5j**).

Quantification of CD98hc expression in endothelial cells and astrocyte endfeet

To quantify the localization of CD98hc within brain vessels, the vessel surface was measured by combining the vascular markers Glut1 and AQP4. First, the individual Glut1 and AQP4 signals were segmented using the Surfaces method in Imaris (v9.9.0, Bitplane). AQP4 was segmented using background subtraction, with the largest object radius set to 0.5 µm, the minimum object intensity after subtraction set to 580 AU, and region growing enabled with the largest region growing diameter set to 0.211 µm, accepting any objects 0.312 μm^3 or larger. Glut1 was segmented using background subtraction, with the object radius set to 0.5 µm, the minimum object intensity after subtraction set to 500 AU, and region growing enabled with the largest region growing diameter set to 0.211 µm, accepting any objects 1.81 μm^3 or larger.

The resulting segmentations were hollow with gaps along the vessel surface that prevented naïve calculation of vessel diameter. To produce a watertight vessel mesh, both surfaces were first converted to a Boolean mask and then downsampled to 0.263 μm isotropic using the transform.rescale function in scikit-image (v0.19.2)(*2*). The Boolean OR of AQP4 and Glut1 signals were calculated, then the vessel surface was smoothed using binary closing. The outer voxels of the vessel were converted to a point cloud and then meshed using the delaunay_3d function in pyvista (v0.37.0)(*3*) with the alpha shape parameter set to 1.0 μm. Holes in the resulting mesh were corrected using the pre.remesh (using voxel size 0.1 μm, adaptivity 0.1) and pre.simplify (using ratio 0.1) functions in skeletor (v1.2.3). The centerline of the vessel mesh was calculated using the skeletonize.by wavefront function in skeletor with 100 waves and a step size of 1, then the skeleton converted to a final centerline using the post.radii function with the k-nearest neighbors method, using 5 nearest neighbors and aggregating on the mean. The instantaneous local distance from the surface to the skeleton midline and vice versa was calculated using the ndimage.distance_transform_edt function in scipy (v1.9.3)(*4*).

Even within a single image, the distance from centerline to surface varied widely between < 1.0 μm to > 5.0 μm. To summarize the location of Glut1, AQP4, and CD98hc, bins between 0.0 μm and 5.0 μm in steps of 0.1 μm were calculated for distance to the vessel surface (0.0 on the vessel surface, 5.0 furthest from the surface) and between 0.0 μm and 5.0 μm in steps of 0.1 μm were calculated for distance from the vessel centerline (0.0 on the vessel centerline, 5.0 μm furthest from the centerline). The total signal in each 2D bin was calculated across n=15 images and then the marginal distribution of AQP4, Glut1, and CD98hc was calculated by averaging across all distance to centerline bins, producing a 1D distribution of signal as measured from the vessel surface inward to the centerline.

Peripheral tissue PK and CD98hc western blots

Peripheral tissues were homogenized and analyzed for huIgG as described under *"Quantification of huIgG"* in main Methods section. For CD98hc western blots: frozen tissues were homogenized using a Qiagen TissueLyser in 10x tissue weight of lysis buffer containing 1% NP-40 + protease and phosphatase inhibitors in PBS. Total protein concentration was measured with BCA and samples were diluted to 1 mg/mL of total protein in NuPAGE LDS sample buffer (4X) with NuPAGE sample reducing agent (10X) and boiled for 10 minutes. Samples were run on

NuPAGE 4-12% Bis-Tris gels. Human CD98hc was detected with rabbit anti-human CD98hc and mouse beta actin as a loading control. Primary antibodies were detected with licor secondaries. See **Supplemental Table 7** for details on primary and secondary antibodies. Blots were imaged on a Li-Cor Odyssey CLx and quantified with Image Studio Lite (Li-Cor, Version 5.2.5).

In vitro cell trafficking

HEK293 cells (ATCC, CRL-1573) were maintained in DMEM (Gibco™ 11995073) + 10%FBS (VWR 89510-188) + 1x Pen/Strep (Gibco 15140122) plated (25,000 cells/well) on 96 well plates (PhenoPlate). Cells were washed with warm HBSS then starved for 1 h in DMEM without serum at 37°C. Molecules were diluted in DMEM and applied to cells at 250 nM for 2 h at 37°C; the relatively high concentrations were designed/optimized to enable detection of huIgG in lysates by ELISA. Cells were then washed in HBSS, incubated in DMEM without serum for two h at 37°C, and washed in PBS before fixation or lysis.

For immunocytochemistry: After the last PBS wash, cells were fixed in cold 4% PFA and stored at 4°C overnight until staining. Cells were blocked in 1% BSA + 0.05% Tween20 in PBS, incubated with molecules in blocking solution overnight, washed twice in PBS, then incubated with secondary antibody (1:1000, Jackson ImmunoResearch 109545), cell mask (1:10000, Thermo C10046), and DAPI (1:2000, Thermo D1306) in 1% BSA in PBS for 1.5 h. Sample plates were imaged on an automated confocal high-content imaging system (Opera Phenix; Perkin Elmer) using a 63X water immersion objective lens with excitation lasers (405 nm, 488 nm; 640 nm) and preset emission filters. Channels were separated to avoid fluorescence crosstalk. For huIgG quantification, a custom protocol was developed in the Harmony 4.9 image analysis software (Perkin Elmer). Briefly, cells were identified using the 'Find Nuclei' building block with the DAPI channel. The cell region was further defined with the Cell Mask channel using 'Find Cytoplasm'. The mean fluorescence intensity of huIgG was measured across the entire cell, calculated on a per-cell basis, and then averaged across the well.

For huIgG detection by ELISA: At time 0 h, media was collected before the last HBSS wash and transferred to a new plate and the cells were lysed for 15 min following application of cold RIPA buffer containing protease and phosphatase inhibitors and benzonase cells. The cells

and buffer were pipetted to mix, and two replicate wells per condition were pooled together and transferred to a new plate. For later time points, at 0 h the media was removed, cells washed in PBS, and fresh media without serum applied before returning cells to the incubator for processing as above at designated time points. The media and lysate plates were frozen until analysis using the huIgG ELISA.

Aβ40 measurements

BACEl inhibition of amyloid precursor protein APP cleavage was used as a pharmacodynamic readout of antibody activity in brain as previously described(*5*). Brain tissue was homogenized in 10x tissue weight of 5 M guanidine-HCl and then diluted 1:10 in 0.25% casein buffer in PBS. Mouse Aβ40 levels in brain lysate were measured using a sandwich ELISA. A 384-well MaxiSorp plate was coated overnight with a polyclonal capture antibody specific for the C-terminus of Aβ40 peptide (Millipore #ABN240). Casein-diluted guanidine brain lysates were further diluted 1:2 on the ELISA plate and added concurrently with the detection antibody, biotinylated anti-mouse/rat β-Amyloid M3.2. Samples were incubated overnight at 4°C prior to addition of streptavidin-HRP followed by TMB substrate. The standard curve, 0.78-50 pg/mL ms Aβ40, was fit using a four-parameter logistic regression.

Mathematical modeling

The mathematical PK model of CD98hc TVs is shown in **Supplemental Figure 13a**. The two-compartment model of the systemic PK includes non-specific and CD98hc-mediated clearances from the systemic circulation. Uptake mechanisms of ATV^{CD98hc} into the brain via low-efficiency non-specific transport as well as high-efficiency CD98hc-mediated transcytosis across brain endothelial cells are also incorporated in the model. The CD98hc-mediated brain uptake and clearance processes are explicitly represented as a function of apparent affinity of the molecule to CD98hc. The apparent affinity is increased for $biATV^{CD98hc}$ compared to their monovalent counterparts. Additionally, the model explores independent parameters to represent the effect of affinity on systemic and brain compartments to account for potential differences in the CD98hc expression level systemically compared to brain endothelial cells. The loss of antibodies from the brain is modeled as a first-order linear elimination process with the rate constant (k_{loss}).

The model was developed using the Simbiology® Toolbox (MATLAB 2022b, MathWorks), and calibration of model parameters was performed using the Scatter Search global

optimization algorithm. Model parameter estimation was performed in primarily two stages of systemic PK calibration followed by brain PK calibration. Pooled analysis was performed to estimate one reference calibration across all datasets for control huIgG and ATV^{CD98hc} molecules. Additionally, molecule specific parameters were introduced based on the mechanistic understanding of biodistribution. For example, increased k_{loss} for anti-BACE1 and ATV^{CD98hc}:BACE1 molecules was introduced to capture their lower brain concentrations compared to their DNP counterparts, and CD98hc-mediated processes were turned off in the model for control huIgG molecules. **Supplemental Table 4** shows the parameter values and **Supplemental Figure 13b-g** demonstrates a representative comparison of the model-based fits to the experimental data across multiple studies in CD98hc$^{mu/hu}$ KI mice.

Despite inherent inter-animal and inter-study variability in the data, representative fits indicate a good agreement between model simulations and ATV concentrations in the systemic circulation and the brain. Since a single parameter set is used to capture all experimental data, model fits are expected to follow the collective trend in the observed values, and as such, mismatches with individual data points, or a small subset of data, are expected and inevitable. Additionally, the coefficient of variation of the mean of the estimated parameters (CV95%) indicate that an acceptable fit to the collective dataset has been achieved (**Supplemental Table 4**).

To explore the hypothesis of ATV^{CD98hc} retention in the brain and to quantify the effect of Fab binding and valency, the molecule specific k_{loss} was estimated in the calibration process. Estimated molecule specific $k_{loss,ATV}$ values as a mean ± SD (across all studies) are plotted for ATV^{CD98hc}:DNP molecules with respect to their corresponding CD98hc affinity (**Supplemental Figure 13h**). Note that data for monovalent ATV^{CD98hc}:DNP molecules with CD98hc K_D of 20 nM was excluded from the analysis due to the observed reduction of brain C_{max} compared to molecules with weaker CD98hc affinity. The estimated $k_{loss,ATV}$ values collectively show a decreasing trend with stronger affinity, consistent with experimental evidence for the affinity dependent retention of ATV^{CD98hc}:DNP in the brain. In addition, at the same CD98hc affinity, $k_{loss,ATV}$ values for biATV^{CD98hc}:DNP are estimated to be lower than monoATV^{CD98hc}:DNP, indicating higher retention due to higher binding valency. $k_{loss,ATV}$ estimates for both monovalent and bivalent ATV^{CD98hc}:DNP molecules are also below the estimated value for the control huIgG ($k_{loss,nonATV}$), further providing evidence for retention of ATV^{CD98hc}:DNP and lower brain loss due to CD98hc binding. The higher retention hypothesis with CD98hc binding, however, cannot justify

lower brain C_{max} observed with $ATV^{CD98hc6.29}$:DNP (20 nM CD98hc K_D) compared to weaker-affinity molecules. This lower C_{max} may indicate potential mechanisms affecting the brain uptake dynamics at CD98hc affinities above a certain threshold. Exploration of such mechanisms is beyond the scope of the current model. Parameter estimates also suggest that different apparent affinities in the systemic and brain compartments may be needed to better capture the PK differences between mono- and $biATV^{CD98hc}$:DNP (**Supplemental Table 4**).

We subsequently analyzed how differences observed in the PK of ATV^{CD98hc}:BACE1 and ATV^{CD98hc}:DNP molecules can be explained using the least possible degrees of freedom. The systemic PK for both monovalent and bivalent ATV^{CD98hc}:BACE1 was consistent with ATV^{CD98hc}:DNP data, indicating negligible effect of BACE1-mediated clearance in the systemic circulation. Consequently, the same systemic PK model parameters were used to capture ATV^{CD98hc}:DNP and ATV^{CD98hc}:BACE1 data (**Supplemental Figure 13b,d**). Given similar circulating concentrations, we further assumed that the non-specific and CD98hc-mediated specific uptake parameters are also shared between the data for both Fabs. As such, we only performed parameter estimation for brain loss rate constants necessary to capture the reduced brain PK data observed in ATV^{CD98hc}:BACE1 and anti-BACE1 molecules.

The analysis (**Supplemental Table 4**) indicates that ~2 fold increase in $k_{loss,nonATV}$ and ~6/~13 fold increase in $k_{loss,ATV}$ for mono- and $biATV^{CD98hc}$:DNP molecules is needed to capture the brain PK data for ATV^{CD98hc}:BACE1 molecules. **Supplemental Figure 13e** demonstrates that the hypothesis of CD98hc-mediated protection explains the differences in brain PK across ATV^{CD98hc}:DNP and ATV^{CD98hc}:BACE1 molecules. Estimated increased brain loss in ATV^{CD98hc}:BACE1 compared to ATV^{CD98hc}:DNP is also consistent with the experimental observation of neuronal internalization of ATV^{CD98hc}:BACE1 molecules, enhancing the degradation compared to non-neuronally localized molecules, i.e., ATV^{CD98hc}:DNP.

Model Equations

$$\frac{dA_1}{dt} = IV\ dose - CL.C_1 - \frac{V_m C_1}{K_{ms} + C_1} - CL_D(C_1 - C_2)$$

$$\frac{dA_2}{dt} = CL_D(C_1 - C_2)$$

$$\frac{dC_b}{dt} = k_{up}^{ns} C_1 + \frac{k_{up}^{CD98hc} C_1}{K_{mb} + C_1} - k_{loss} C_b$$

$$C_1 = \frac{A_1}{V_1},\ \ C_2 = \frac{A_2}{V_2}$$
$$K_{ms} = \ \alpha f_s\, K_D$$
$$K_{mb} = \ \alpha f_b\, K_D$$
$$k_{loss} = \begin{cases} k_{loss,nonATV} & non\ ATV \\ k_{loss,ATV} & CD98hc\ TVs \end{cases}$$
$$Brain\ uptake = \ k_{up}^{ns} C_1 + \frac{k_{up}^{CD98hc} C_1}{K_{mb} + C_1}$$
$$Brain\ clearance = \ k_{loss} C_b$$
$$Net\ Brain\ Flux = Brain\ uptake - Brain\ clearance$$

Human brain microvessel proteomics

All brain tissues samples were provided by University of Kentucky – Alzheimer's Disease Center (UK-ADC) Brain Bank. For each subject we performed analysis on brain tissue dissected from the medial frontal cortex and the cerebellum

Vessels Isolation Procedure

A protocol for isolation of human brain vessels was adapted from previously described protocol for mouse brain vessel purification(*6*). Human brain samples were thawed in B1 buffer for 15 min, cut into smaller pieces and homogenized using an automated Dounce homogenizer at 400rpm. The resulting pellet was resuspended by shaking in dextran buffer B2 and centrifuged at 4400g for 15 min at 4°C. The white myelin layer and supernatant were discarded, and any residual myelin on the sides of the tube was removed with a Q-tip. The pellet containing the blood vessels was resuspended in B1 buffer and passed through a glass-bead column. The column consisted of 5g of glass beads (Sigma G9268) placed on a 100 µm filter and was pre-equilibrated with B1 buffer. The vessels adhering to the glass beads were collected by washing the beads in a buffer containing BSA (B3 buffer). To enrich for capillaries, vessel solution was passed through a 70 µm-mesh filter followed by a 20 µm-mesh filter. Vessels captured by the 20 µm filter were washed in ice cold PBS, subsequently frozen on dry ice and transferred to -80°C until further proteomics analysis.

Sample Preparation for Mass-Spectrometry

To maximize detection of membrane proteins, a non-ionic lysis buffer ((HEPES 50 mM, pH 8.5); sodium chloride (100 mM); DDM (1% w/v), sodium fluoride (10 mM), β-glycerophosphate (10 mM), sodium orthovanidate (2 mM); sodium pyrophosphate (10mM), in

HPLC grade water)) was used for tissue preparation. Lysis buffer was added to frozen capillaries. Homogenization was carried out by probe sonication on ice, using 3 cycles at 25% power (10 sec on followed by 15 sec off). Urea (600 µL, 8 M in 50 mM HEPES, pH 8.5) was added to the homogenate with gentle vortexing. The resultant proteins were reduced and alkylated(*7*). Proteins were precipitated by addition of 1 volume (100% w/v) trichloroacetic acid to 4 volumes of sample, followed by cooling to 4°C and centrifuging 18000 rcf for 5 minutes. Precipitated proteins were dried at 56°C, before being re-dispersed in 300 µL of 1 M urea in 50 mM HEPES pH 8.5. Digestion was then carried out, first by LysC (addition of 15 µL of 0.2 µg/µL solution with vortexing at room temperature for 8 hours), then by trypsin (7.5 µL of 0.4 µg/µL at 37 °C, for 6 hours). Digestion was quenched by adding 20 µL of 10% trifluoroacetic acid. Resultant peptides were de-salted(*8*) before being labeled with 10-plex TMT reagents (Thermo Scientific, 90110)(*9*). Labeled samples were pooled then fractionated using a Pierce™ High pH Reversed-Phase Peptide Fractionation Kit (cat 84868). Fractions were dried under vacuum and re-suspended in 5% formic acid/5% acetonitrile and analyzed by liquid chromatography (LC)-MS^2/MS^3 for identification and quantitation.

LC-MS2/MS3 Analysis

All LC-MS^2/MS^3 experiments were performed as described previously(*10*). Analysis was performed on an Orbitrap Fusion mass spectrometer equipped with an Easy-nLC 1000, using a home packed column (inner diameter, 100 µm; outer diameter, 360 µm). Columns were packed first with ~0.5 cm of C_4 resin (5 µm, 100 Å) followed by ~0.5 cm of C_{18} resin (3 µm, 200 Å) and then to a final length of 30 cm with C_{18} (1.8 µm, 120 Å). Peptides were eluted with a linear gradient (11 to 30% acetonitrile in 0.125% formic acid over 165 min at a flow rate of 300 nL/minute) with column heating at 60C. Electrospray ionization voltage was set to 2000V. Mass spectrometer operating conditions included collection in data-dependent mode, with a survey scan over a mass to charge (*m*/*z*) range of 500–1200 at a resolution of 120,000 in the Orbitrap. Automatic gain control (AGC) was set to 5×10^5 for the MS1 survey scan, with a maximum injection time of 100ms and the s-lens set to an RF of 60. The most abundant ions observed in the survey scan were subjected to MS2 and MS3 analysis for identification and quantitation respectively. Top Speed mode was used acquiring a maximum number of spectra in a 5 s experimental cycle. Data collected at MS1, MS2, and MS3 levels were centroided.

For MS2 analysis, ions above an intensity threshold of 5 × 10^5 were isolated in the quadrupole portion of the mass spectrometer (isolation window of 0.5 *m*/*z)*, then fragmented with collision-induced dissociation with a normalized energy of 30%. Fragment ions were detected with rapid scan rate setting enabled in the ion trap, with an AGC setting of 1 × 10^4 and maximum injection time of 35 ms. MS3 analysis for quantification of TMT reporter ions was performed using the synchronous precursor selection setting(*9*). A maximum of 10 MS2 ions were simultaneously isolated and fragmented for MS3 analysis. An isolation window of 2 *m*/*z* was utilized to isolate MS2 fragments, with further fragmentation by high energy collision dissociation at a normalized energy of 50%. Fragment ions were detected in the Orbitrap at a resolution of 60,000 with a low mass of 110 *m*/*z* for MS3 analysis. The MS3 AGC was set to 5 × 10^4 with a maximum ion injection time of 150 ms. MS2 ions in the range of 40m/z below and 15 *m*/*z* above the precursor (MS1) were excluded from selection for MS3.

Data Processing and Analysis

Data were processed using the ProteomeDiscoverer 2.1.0.81 software package equipped with the built-in version of SequestHT. Identities were assigned to MS2 spectra by searching against the Uniprot Human database (downloaded Feb. 6, 2019). Search parameters include a 50ppm MS1 mass tolerance, 0.6 Da fragment ion tolerance, fully-enzymatic trypsin with a maximum of two missed cleavages per peptide, static modifications of 10-plex TMT tags on lysines and peptide n-termini and carbamidomethylation of cysteines. Variable modifications included oxidation of methionines and phosphorylation of serine, threonine and tyrosine residues. Data were filtered to a peptide and protein false discovery rate of less than 1%. Peptides matching to multiple proteins were assigned to the protein containing the largest number of matched redundant peptides. TMT reporter ion intensities were extracted from MS3 spectra for quantitative analysis where spectra were required to have greater than 10 average signal-to-noise per label and isolation interference of less than 25%(*11*). Data were normalized in a multistep process, whereby they are first normalized to the median of the bridge channels (each abundance is divided by the ratio of its corresponding bridge channel value to the median value of both bridge channels). Data are then then normalized to the average for each peptide, and to the median of all averages. Finally to account for slight differences in amounts of protein labeled, these values are then normalized to

the median of the entire data set and reported as final normalized signal-to-noise ratios per peptide per sample. A tutorial and corresponding R script were previously published(*10*).

a

Fc EU numbering	380	381	382	383	384	385	386	387	422	423	424	425	426	438	439	440
	E	W	E	S	N	G	Q	P	V	F	S	C	S	Q	K	S
**Front.1**	NHK		NNK		NHK	NNK	NHK	NNK								
**Front.2**	NNK		NHK		NNK	NHK	NNK	NHK								
**Front.3**	NHK		NHK		NHK	NHK	NHK	NHK								
**Back.1**									NNK		NHK		NNK	NHK		NNK
**Back.2**									NHK		NNK		NHK	NNK		NHK
**Back.3**									NHK		NHK		NHK	NHK		NHK

b

Fc EU numbering	380	381	382	383	384	385	386	387	422	423	424	425	426	438	439	440	Human/ Cyno cross-reactivity
	E	W	E	S	N	G	Q	P	V	F	S	C	S	Q	K	S	
TV1	D		R		Y	Y	T	R	K		V		D	I		T	no
TV2	D		R		Y	F	P	F	L		L		D	V		P	no
TV3	D		N		Y	L	F	T	I		W			F		K	no
TV5	L		N		R	F	F	I	I		A		N	F		N	yes
TV6	L		N		K	F	E	L	L		A		N	F		N	yes

Supplemental Figure 1. Naive β-sheet library designs and naïve CD98hc-binding TVs hits. a. Naïve β-sheet library designs are shown aligned to the wild-type Fc sequence. Three "front" and "back" fragments were generated using different randomization strategies and were combined to create nine pooled initial TV libraries. NNK encodes for all twenty amino acids, whereas NHK restricts diversity by excluding glycine, cysteine, tryptophan, and arginine. **b.** The sequences of the five initial TV hits to CD98hc are shown with their human and cynomolgus CD98hc cross-reactivity noted.

a

Name	Type	380	382	384	385	386	387	422	424	426	428	434	438	440
AM1	AA	X	X	X	X	X	X	LIV	A	N	ML	NS	F	N
	codons	NNK	NNK	NNK	NNK	NNK	NNK	VTH	GCC	AAT	MTG	ARY	TTT	AAT
AM2	AA	L	N	KRNS	F	EFYDV	LIVF	X	X	X	ML	NS	X	X
	codons	CTG	AAT	ARN	TTT	KWN	NTY	NNK	NNK	NNK	MTG	ARY	NNK	NNK

X = any amino acid

b

Name	Type	378	380	382	383	384	385	386	387	389	391
AM3	AA	Z	L	N	Z	R	F	VASL	L	Z	Z
	Codons	NHK	CTG	AAT	NHK	CGC	TTT	KYR	CTG	NHK	NHK
AM4	AA		L	Z	Z	Z	F	Z	L		
	Codons		CTG	NHK	NHK	NHK	TTT	NHK	CTG		

Continued:

Name	Type	421	422	424	426	428	436	438	440	441	442
AM3	AA	Z	I	A	N	L	Z	F	N		Z
	Codons	NHK	ATC	GCC	AAT	CTG	NHK	TTT	AAT		NHK
AM4	AA		I	A	N	Z		F	N	LP	Z
	Codons		ATC	GCC	AAT	NHK		TTT	NHK	CYN	NHK

Z = All amino acids <u>except</u> Arg, Trp, Gly, Cys

c

	380	382	383	384	385	386	387	389	390	421	422	424	426	428	436	438	440	442
WT Fc	E	E	S	N	G	Q	P	N	N	N	V	S	S	M	Y	Q	S	S
TV6.8	L	N	S	R	F	V	L	N	N	E	I	A	N	Y	Y	F	N	A
Patch.1		NNK								NNK	NNK						NNK	
Patch.2			NNK	NNK	NNK	NNK												
Patch.3			NNK			NNK	NNK	NNK	NNK									
Patch.4															NNK	NNK	NNK	NNK
Patch.5		NNK		NNK								NNK	NNK					
Patch.6			NNK					NNK		NNK					NNK			NNK
Patch.7		NNK	NNK	NNK								NNK						
Patch.8				NNK		NNK					NNK				NNK			NNK
Patch.9					NNK						NNK					NNK	NNK	
Patch.10			NNK										NNK				NNK	NNK

Supplemental Figure 2. TV library designs used for affinity maturation (AM). a-b. Randomization strategy for AM1 and AM2 (**a**), and AM3 and AM4 (**b**) maturation libraries. Codon usage and resulting amino acid (AA) diversity are shown. X indicates all 20 amino acids, and Z represents all amino acids except for arginine, tryptophan, glycine, and cysteine. **c.** Set of ten patch library designs based off the parental TV6.8 clone and aligned to the wild-type Fc sequence. Positions randomized to all 20 amino acids are indicated by the NNK codon.

Supplemental Table 1: Clearance values of ATV^{CD98hc}:DNP variants in WT mice. Plasma clearance values obtained after a single 10 mg/kg IV dose of ATV^{CD98hc}:DNP variants in WT mice.

TA	Range of CL in WT mice (mL/d/kg)	huCD98hc affinity (nM)
monoATV$^{CD98hc.6.8}$:DNP EF-	7.73 ± 2.96	170
biATV$^{CD98hc.6.8}$:DNP EF-	7.11 ± 1.32	170
monoATV$^{CD98hc.6.8}$:DNP EF+	8.43 ± 1.16	170
monoATV$^{CD98hc.6.29}$:DNP EF+	2.45 ± 1.15	20
monoATV$^{CD98hc.6.38}$:DNP EF+	9.36 ± 4.75	43
monoATV$^{CD98hc.6.39}$:DNP EF+	6.48 ± 1.44	94
monoATV$^{CD98hc.6.d3}$:DNP EF+	12.1 ± 3.7	275
monoATV$^{CD98hc.6.d6}$:DNP EF+	13.1 ± 5.5	550
biATV$^{CD98hc.6.d3}$:DNP EF+	8.93 ± 1.73	275
biATV$^{CD98hc.6.d6}$:DNP EF+	10.4 ± 1.6	550
biATV$^{CD98hc.6.d12}$:DNP EF+	7.49 ± 1.30	2100
Anti-DNP (neg control)	5.62 – 7.34 (range from 3 studies)	-

Supplemental Table 2: TV^{CD98hc}:CD98hc crystal structure information

	TV6.6:CD98hc (PDB: 8G0M)
**Data collection**	
Space group	$C222_1$
Cell dimensions	
a, *b*, *c* (Å)	136.15, 167.13, 83.68
α, β, γ (°)	90, 90, 90
Resolution (Å)	44.7-2.25 (2.32-2.25)
R_{sym}	0.054 (0.994)
$I / \sigma I$	22.1 (2.0)
Completeness (%)	100 (100)
Redundancy	7.4 (7.2)
**Refinement**	
Resolution (Å)	44.7-2.25
No. reflections	45,610
R_{work} / R_{free}	0.20/0.24
No. atoms	5,193
Protein	4,894
Ligand/ion	143
Water	156
B-factors	
Protein	65.44
Ligand/ion	71.87
Water	53.59
R.m.s. deviations	
Bond lengths (Å)	0.0085
Bond angles (°)	1.49

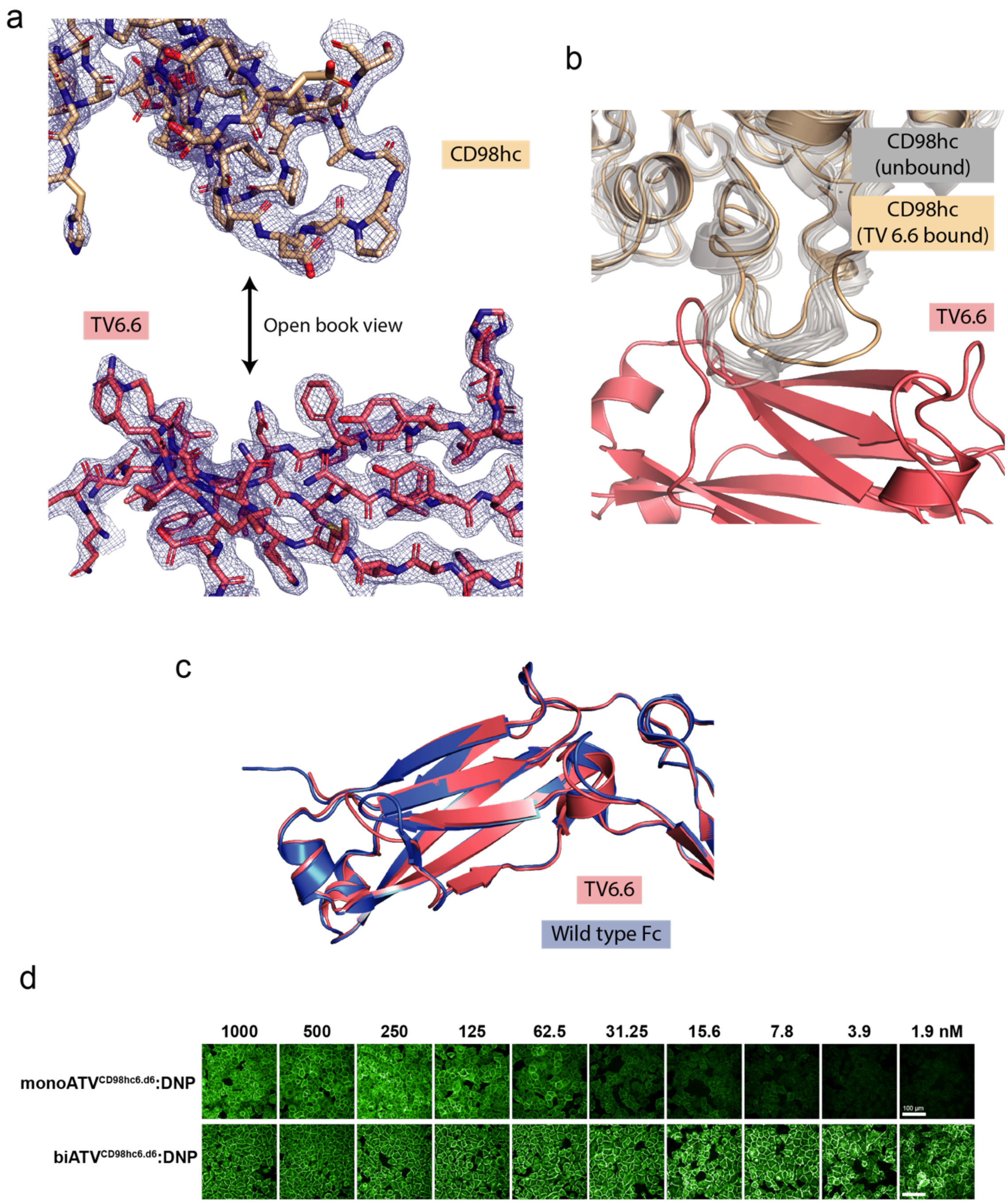

Supplemental Figure 3: Detailed analysis of the TV6.6-CD98hc structure. a. Open book view of the TV6.6 (pink, bottom):CD98hc (tan, top) interface, with electron density shown (blue mesh, 2mFo-DFc contoured at 1.5σ). **b.** Overlay of TV6.6 (pink) bound to CD98hc (tan) with selected CD98hc structures (grey), showing deflection of the CD98hc S495-S504 loop upon TV6.6 binding. (CD98hc PDB IDs = 2DH2, 2DH3, 6IRS, 6IRT, 6JMQ, 6JMR, 6S8V, 7B00, 7DSK, 7DSL) **c.** Overlay of TV6.6 (pink) with wild-type Fc (blue, PDB ID: 4W4O), with Cα RMSD = 0.415Å. **d.** Cell binding of mono- and biATV$^{CD98hc6.d6}$:DNP (550 nM K_D) to HeLa cells show an increased apparent affinity for bivalent CD98hc binding. Scale bars = 100 µm. Representative images are shown from at least 15 images per well, n=2 independent experiments.

a

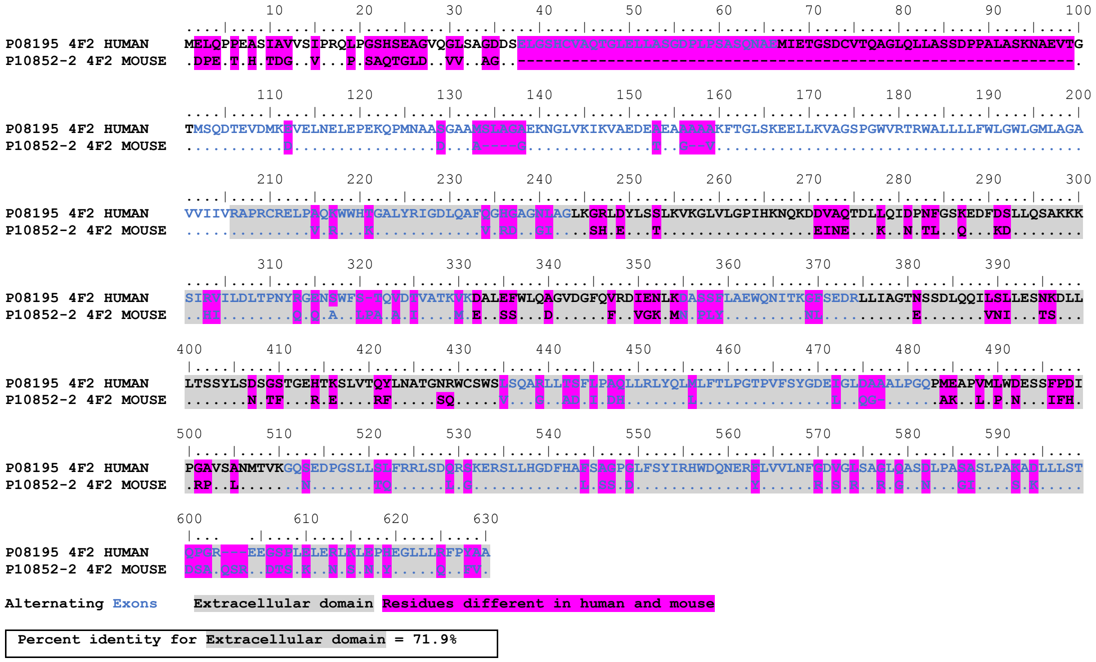

b

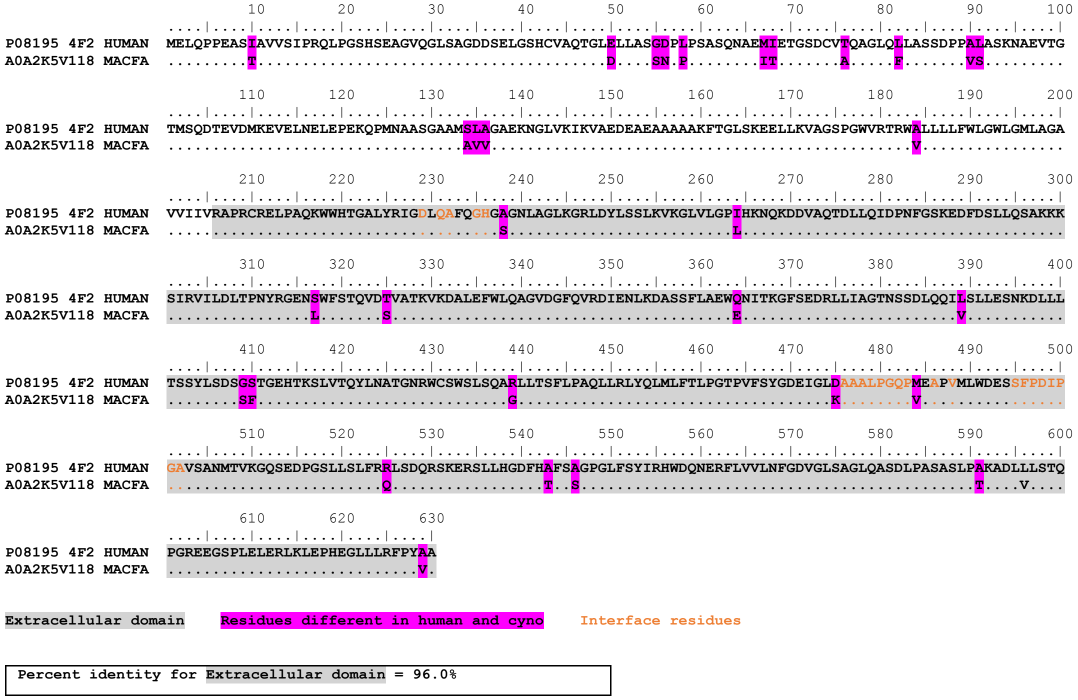

Supplemental Figure 4: CD98hc protein sequence alignments. a. Alignment of human and mouse CD98hc sequences with exons colored alternating in black and blue text. **b.** Alignment of human and cynomolgus CD98hc sequences with positions involved in TV6.6-CD98hc binding colored orange. Percent identity for each alignment is given. The extracellular domain is highlighted in gray and residues that are identical to the human sequence are indicated by a dot. Positions that differ between orthologs are highlighted in magenta.

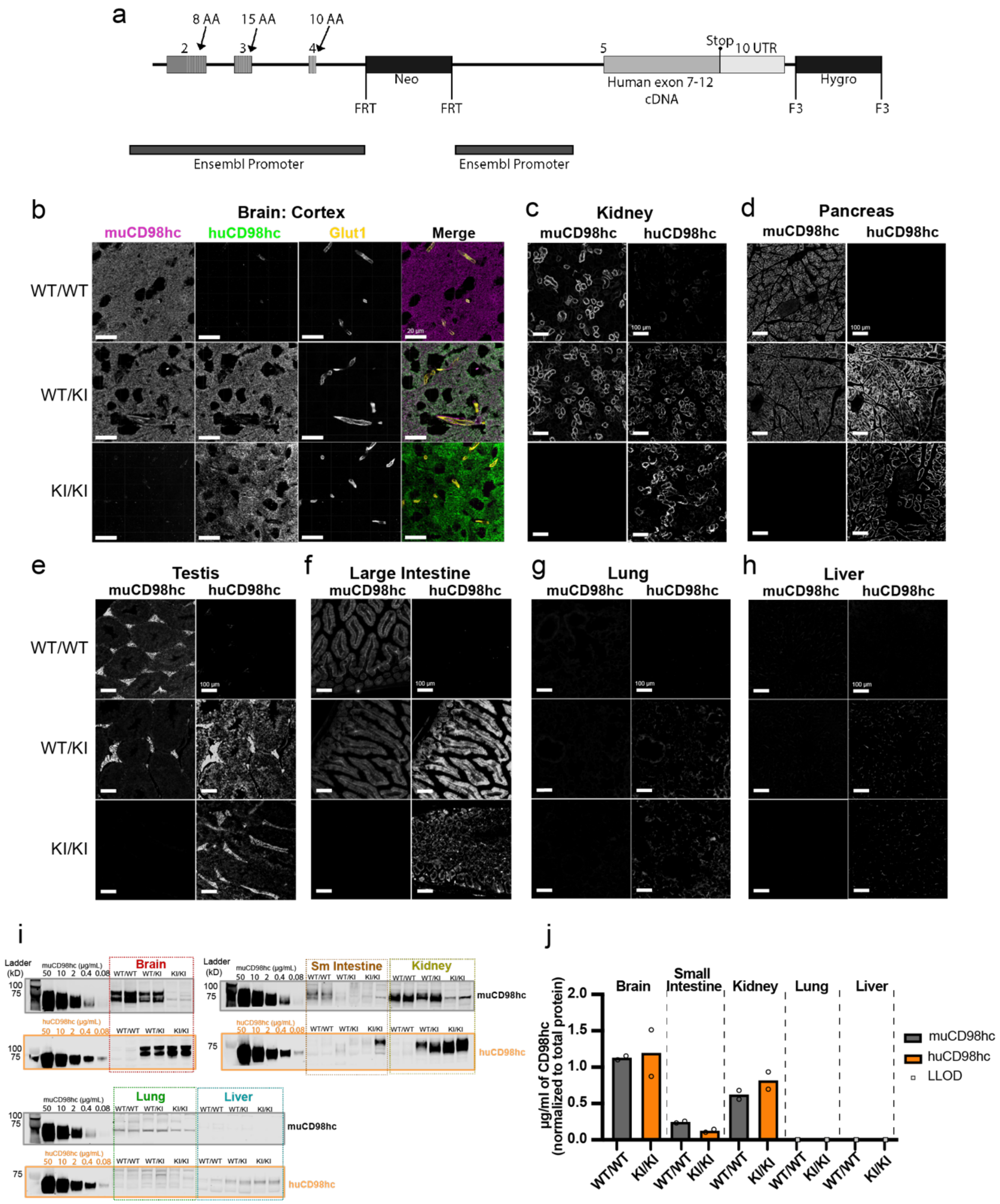

Supplemental Figure 5: Generation and characterization of CD98hc$^{mu/hu}$ KI mice. a. Knock-in construct design to humanize the extracellular domain of CD98hc. **b.** Brain tissue from WT, heterozygous KI, and homozygous KI mice immunostained for CD98hc and Glut1 as a marker for brain vasculature. Scale bars = 20µm. **c-h** Immunohistochemistry staining for CD98hc on peripheral tissues from WT, heterozygous KI, and homozygous KI mice. Representative images for b-h are shown from n=2/genotype (1 male and 1 female). Scale bars = 100µm. **i-j** Western blot and quantification for CD98hc from WT, heterozygous KI, and homozygous KI mice (n=2; 1 male and 1 female). Source data are provided as a Source Data file.

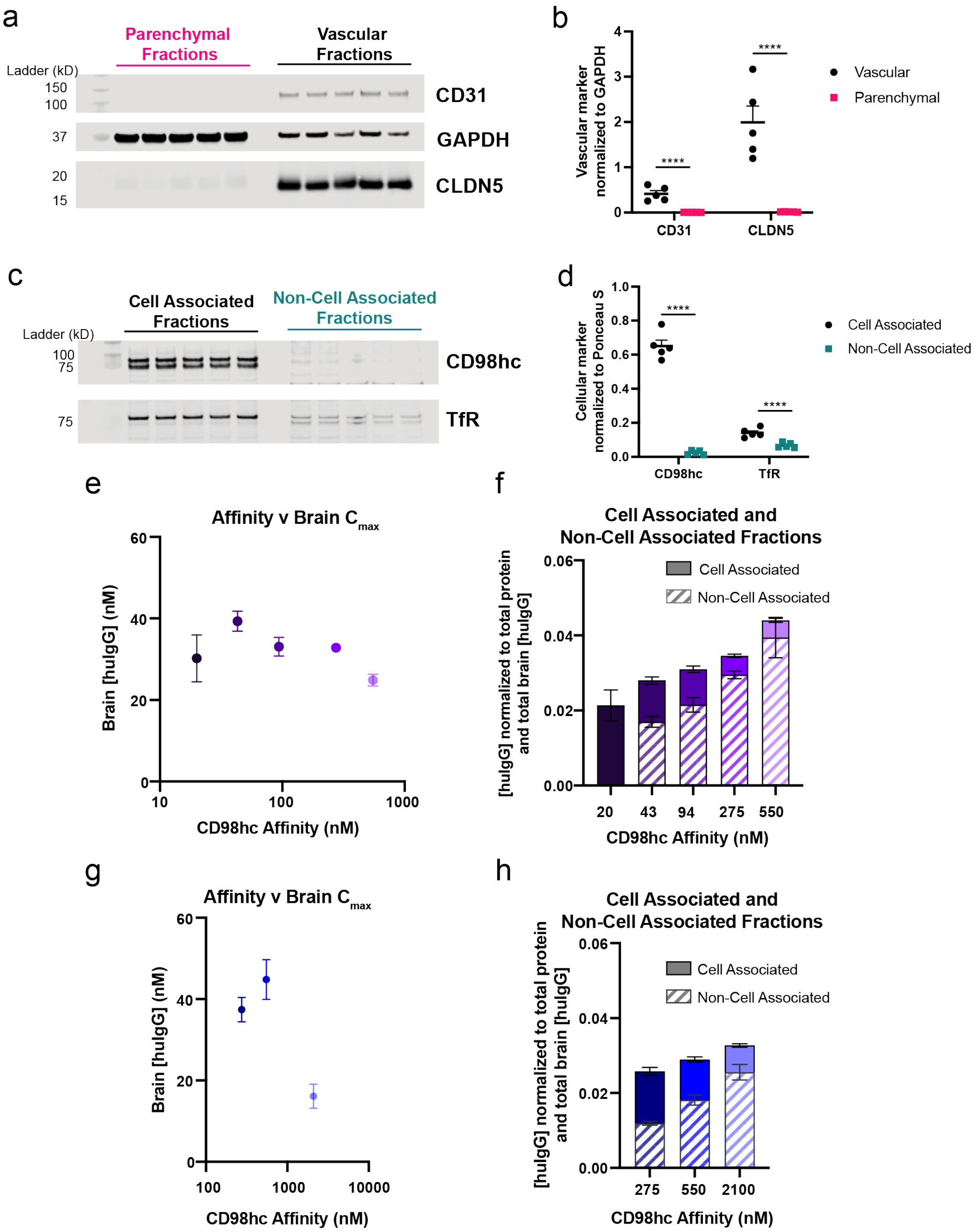

Supplemental Figure 6: Brain uptake and distribution of ATV^{CD98hc}:DNP variants in CD98hc$^{mu/hu}$ KI mice. a-b. Western blots for the endothelial cell markers CD31 and CLDN5, and quantification demonstrating separation of the vascular cells from the parenchymal fraction in mouse. **c-d**. Western blots and quantification of CD98hc and TfR, demonstrating depletion of cell associated proteins from the non-cell associated fraction in mouse. Two-tailed t-test ****= p<0.0001. See Supplemental Table 5 for exact p values. **e.** Brain concentrations of

monoATV^{CD98hc}:DNP clones ranging in affinity from 20-550 nM (ATV$^{CD98hc.6.29}$: 20 nM, ATV$^{CD98hc.6.38}$: 43 nM, ATV$^{CD98hc.6.39}$: 94 nM, ATV$^{CD98hc.6.d3}$: 275 nM, and ATV$^{CD98hc.6.d6}$: 550 nM) 7 days post 50 mg/kg IV dose. **f.** Concentrations of monoATV^{CD98hc}:DNP in cell associated and non-cell associated fractions demonstrating a correlation between stronger affinity and increased cell association (Pearson correlation: r^2=0.98) **g.** Brain concentrations of biATV^{CD98hc}:DNP clones ranging in affinity from 275-2100 nM (ATV$^{CD98hc.6.d3}$: 275 nM, ATV$^{CD98hc.6.d6}$: 550 nM, and ATV$^{CD98hc.6.d12}$: 2100 nM) measured 7 days post single 50 mg/kg IV dose. **h.** Concentrations of biATV^{CD98hc}:DNP in cell associated and non-cell associated fractions demonstrating a correlation between stronger affinity and increased cell association (Pearson correlation: r^2=0.89). **a,d e-h.** n= 4-5/group (see Source Data for exact n/group), graphs display mean ± SEM. Source data are provided as a Source Data file.

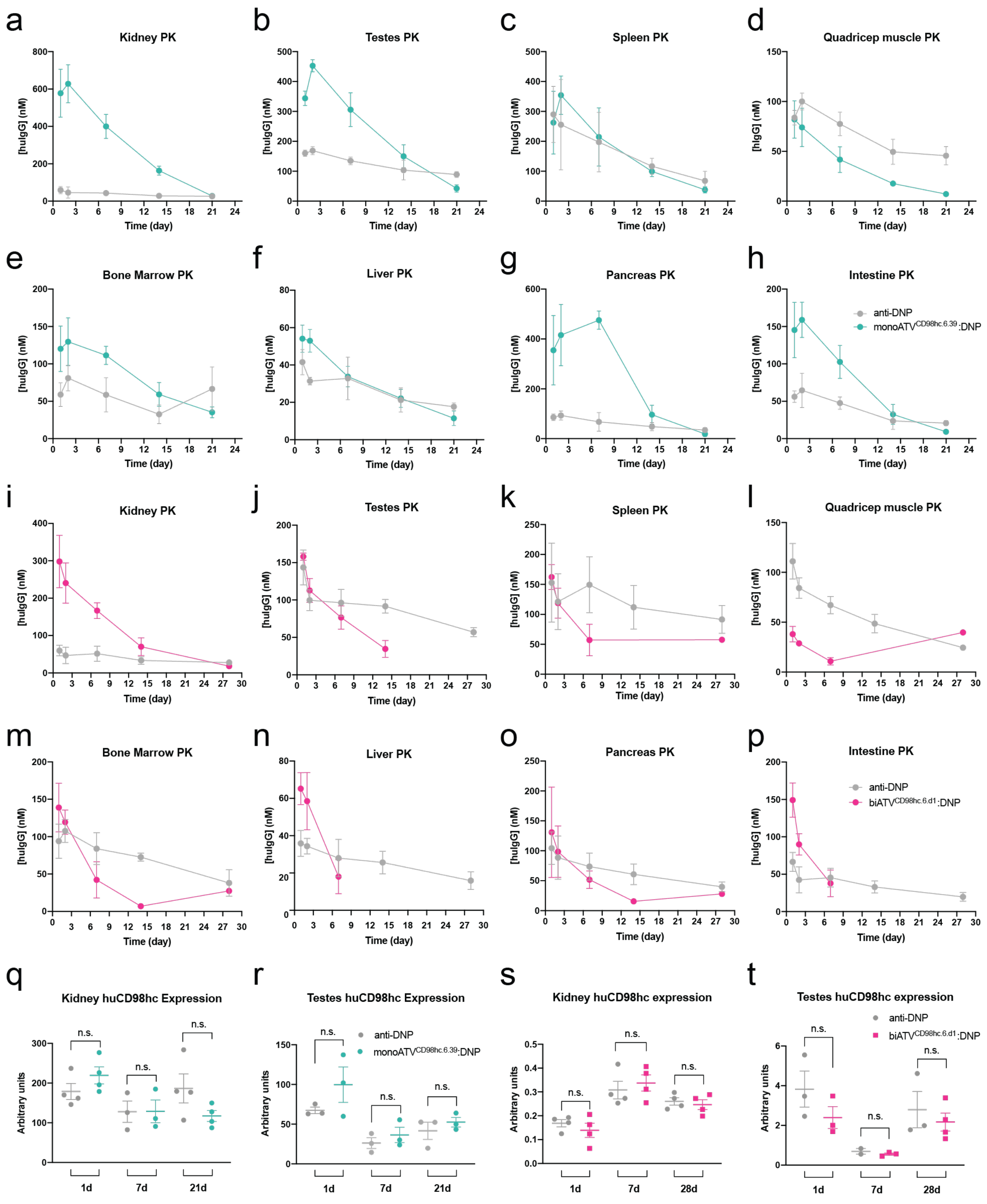

Supplemental Figure 7: Peripheral PK of mono- and biATV^{CD98hc}:DNP. **a-p.** Peripheral tissue concentrations of anti-DNP, monoATV$^{CD98hc.6.39}$:DNP (94 nM K_D), or biATV$^{CD98hc.6.d1}$:DNP (165 nM K_D) after a single 50 mg/kg IV dose. Graphs represent mean ± SD, see Source Data file for n/group for each time point and details on missing data points. **q-t.** Western blot quantification of

CD98hc protein expression normalized to beta actin in kidney (q, s) and testes (r, t) following a single dose 50 mg/kg IV dose of the indicated molecules at 1, 7 and 21 days post-dose. Graphs represent mean ± SEM, unpaired two-tailed t-test. Source data are provided as a Source Data file.

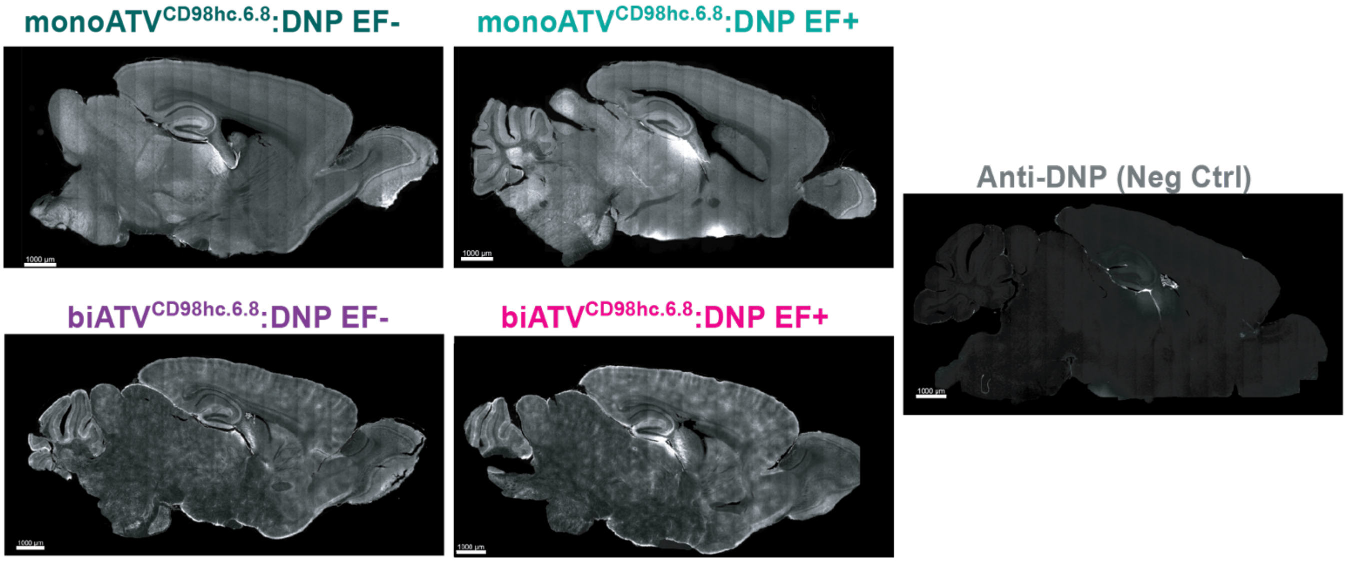

Supplemental Figure 8: Low magnification imaging of ATV^{CD98hc} variants shows broad parenchymal distribution. Mouse brain sagittal sections immunostained for huIgG 7 days post single 50 mg/kg IV dose of ATV$^{CD98hc6.8}$:DNP (170 nM K_D) variants. Representative images are shown from n=5 animals/group, n=2 IHC sections/animal. Scale bars = 1000µm.

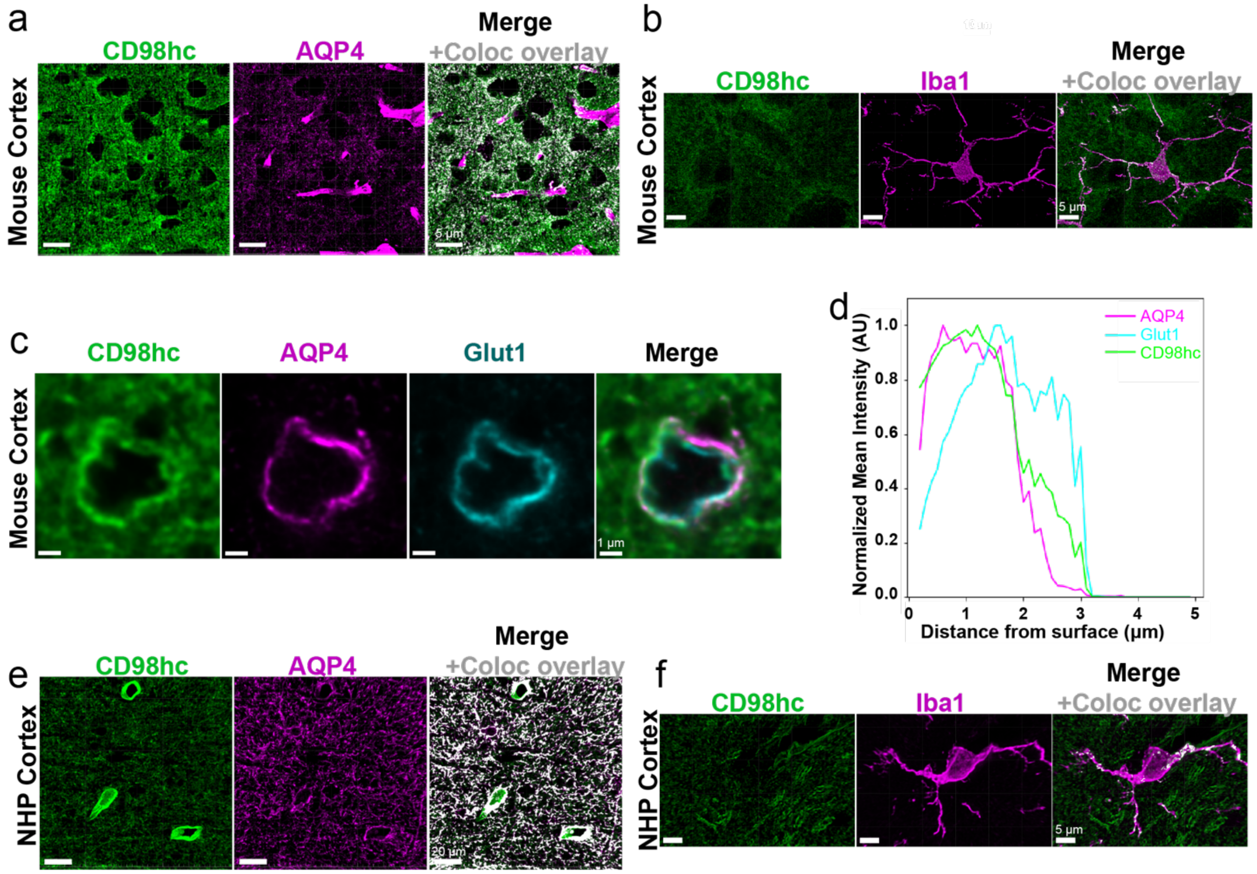

Supplemental Figure 9. Expression of CD98hc in the CNS of mouse and cynomolgus monkey. a-b. Mouse cortical brain sections immunostained for CD98hc (green) and AQP4 (a, purple) or Iba1(b, purple). Representative images are shown from n=5 animals/group, n=2 IHC sections/animal. Scale bars = 5 µm **c.** High magnification super-resolution confocal imaging of representative mouse brain vessels co-stained for CD98hc (green), AQP4 (purple), and Glut1 (aqua) demonstrating CD98hc expression on both brain endothelial cells and astrocyte endfeet. Representative images are shown from n=4 animals/group, n=2 IHC sections/animal. Scale bars = 1 µm **d.** Quantification of co-colocalization of CD98hc with Glut1 and AQP4. **e-f.** Cynomolgus monkey cortical brain sections immunostained for CD98hc (green) and AQP4 (e, purple, scale bars = 20 µm) or Iba1 (f, purple, scale bars = 5 µm). Representative images are shown from n= animals/group, n=3 IHC sections/animal. Overlays are shown with colocalization pseudocolored in white.

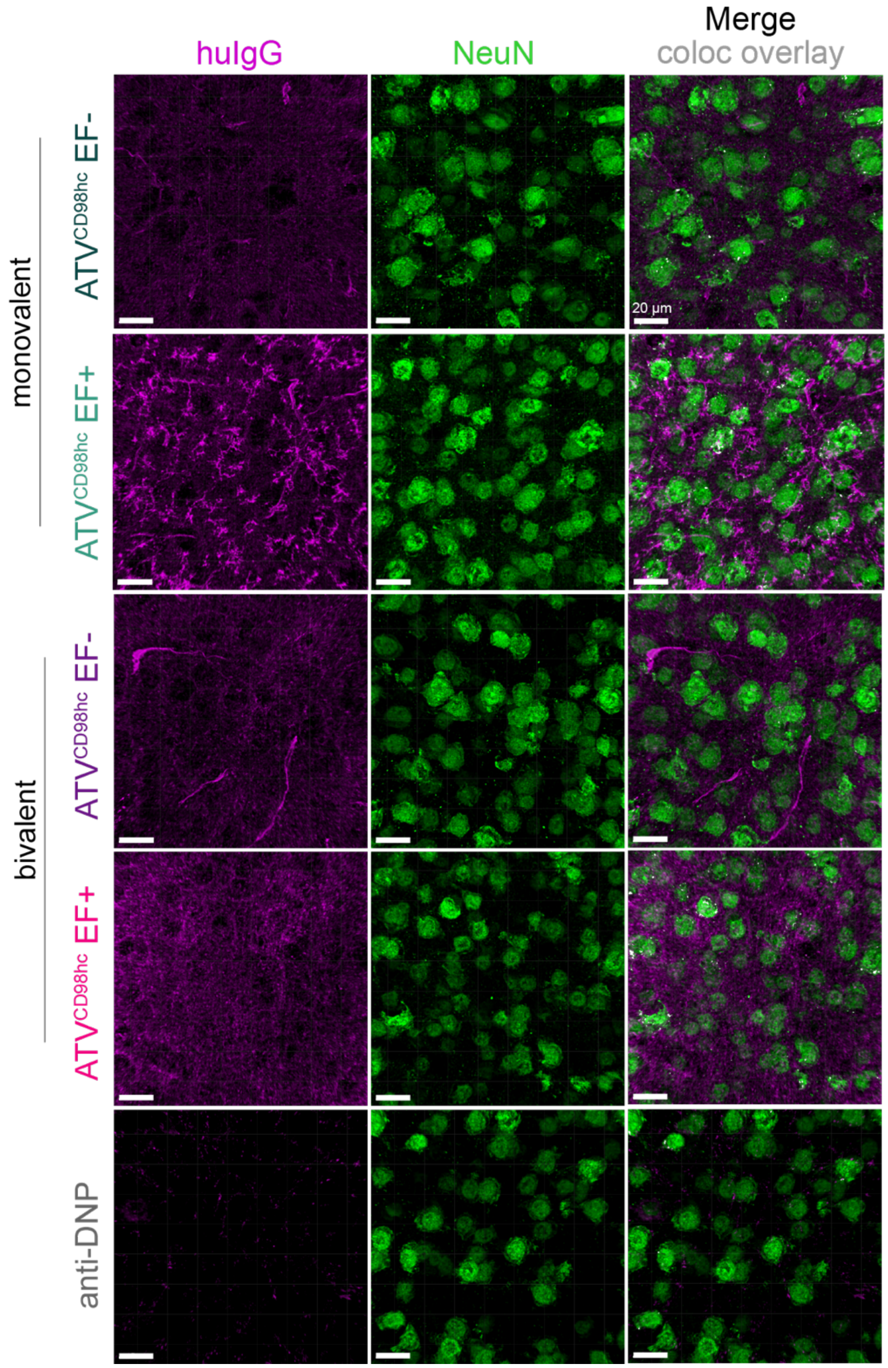

Supplemental Figure 10: IHC showing lack of colocalization of ATV^{CD98hc} variants with neuronal marker. a. Mouse brain sections immunostained for huIgG (purple) and NeuN (green) after a 50 mg/kg IV dose of ATV^{CD98hc} variants (170 nM K_D). Overlays are shown with colocalization pseudocolored in white. Representative image are shown from n=5 animals/group, n=2 IHC sections/animal. Scale bars = 20μm.

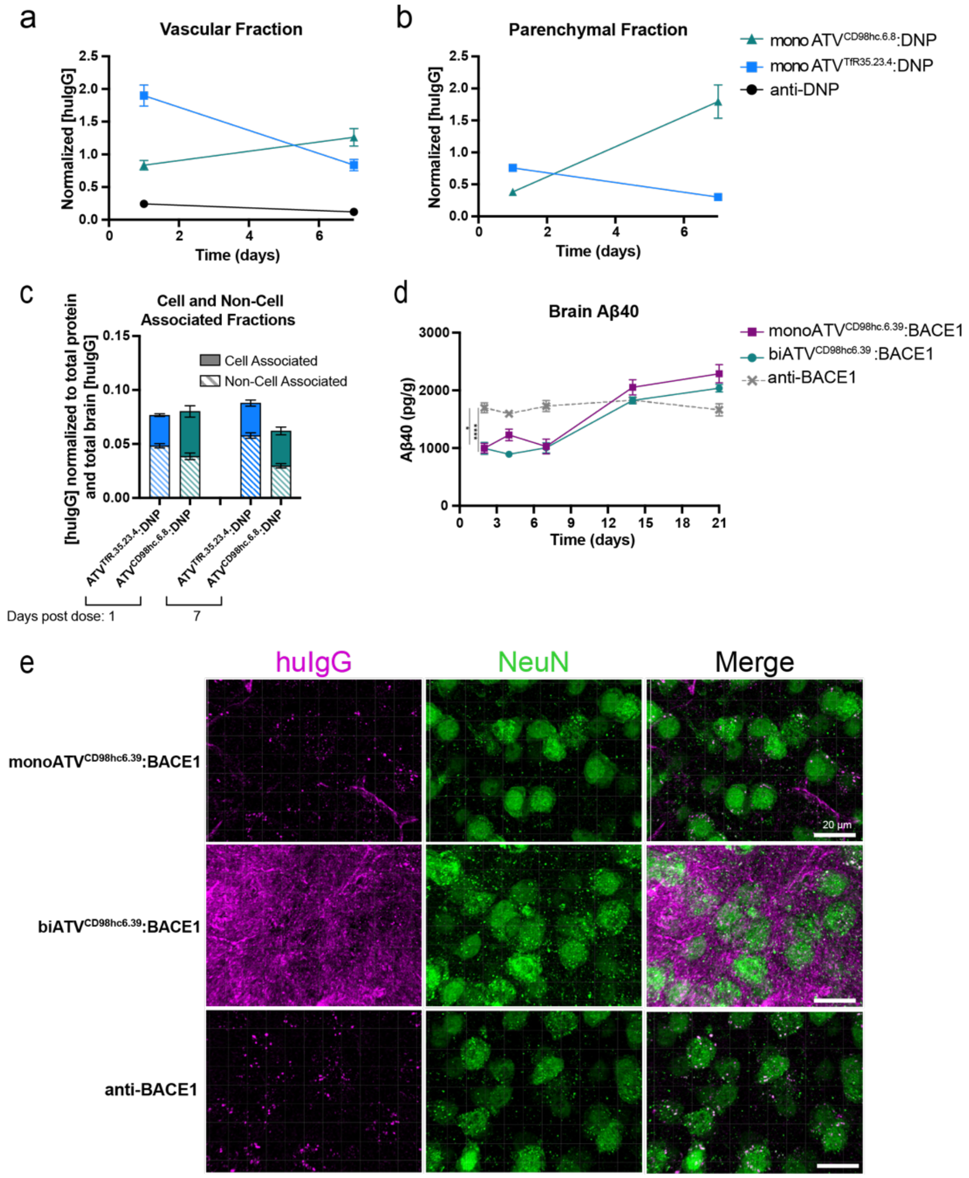

Supplemental Figure 11: Brain uptake and distribution of ATV$^{CD98hc.6.8}$:DNP and ATV^{TfR}:DNP in CD98hc$^{mu/hu}$ KI, TfR$^{mu/hu}$ KI mice. a-c. Concentrations of huIgG in brain vascular (**a**) parenchymal (**b**), cell-associated and non-cell associated (**c**) fractions obtained by capillary depletion following a single 50 mg/kg IV dose of ATV$^{CD98hc.6.8}$:DNP (170 nM K_D) and ATV$^{TfR35.23.4}$:DNP (620 nM K_D). Graphs represent mean ± SEM, see Source Data file for n/group for (**a-b**); n=5/group for (**c**). **b.** Concentrations of anti-DNP were <LLOD in the parenchymal fraction. **d.** Endogenous mouse Aβ40 levels measured in whole brain after a single 50 mg/kg IV dose of mono- or biATV$^{CD98hc6.39}$:BACE1 (94 nM K_D). Two-way ANOVA. *= $p<0.05$ and ****= $p<0.0001$. See Supplemental Table 6 for exact p values. Graphs represent mean ± SEM, n=5/group. **e.** Mouse brain sections immunostained for huIgG1 (purple) and NeuN (green) following a single 50 mg/kg IV dose of ATV^{CD98hc} variants. Representative images shown from n=5 animals/treatment group, n=2 IHC sections per animal. Scale bars = 20 µm. Source data are provided as a Source Data file.

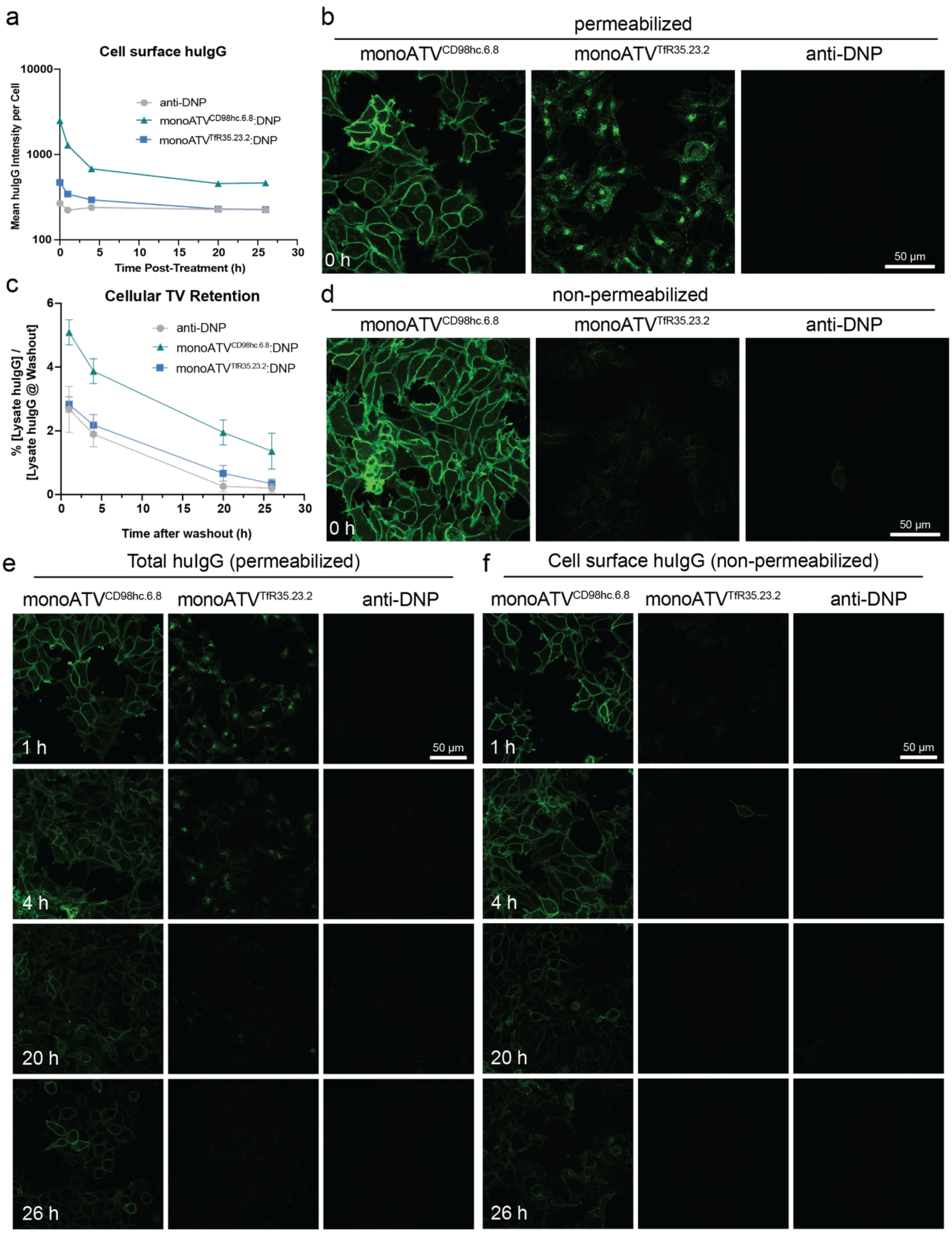

Supplemental Figure 12: Trafficking of ATV^{CD98hc} and ATV^{TfR} in HEK293 cells. a. Quantification of immunocytochemical detection of huIgG in non-permeabilized HEK293 cells immediately following a 2.5 h incubation with 250 nM of monoATV^{CD98hc}:DNP (170 nM K_D),

monoATV^{TfR}:DNP (110 nM K_D), or anti-DNP and washout at 0 h. See Source Data file for number of cells analyzed per time point for n=1 independent experiment. **b.** Representative images of cell surface huIgG immediately after washout (0 h) and later time points **(e)** in non-permeabilized cells. **c**. At designated time points following a 2.5 h incubation with 250 nM of monoATV^{CD98hc}:DNP, monoATV^{TfR}:DNP, or anti-DNP and washout at 0 h, the percent huIgG concentrations in cell lysates of permeabilized HEK293 cells were normalized to the starting concentration at washout (0 h), as measured by ELISA. Graphs represent mean ± SEM n= 5 technical replicates/ time point. **d**. Representative images of immunocytochemical detection of total huIgG immediately after washout (0 h) and later time points (**f**) in permeabilized cells. Scale bars = 50 µm. Source data are provided as a Source Data file.

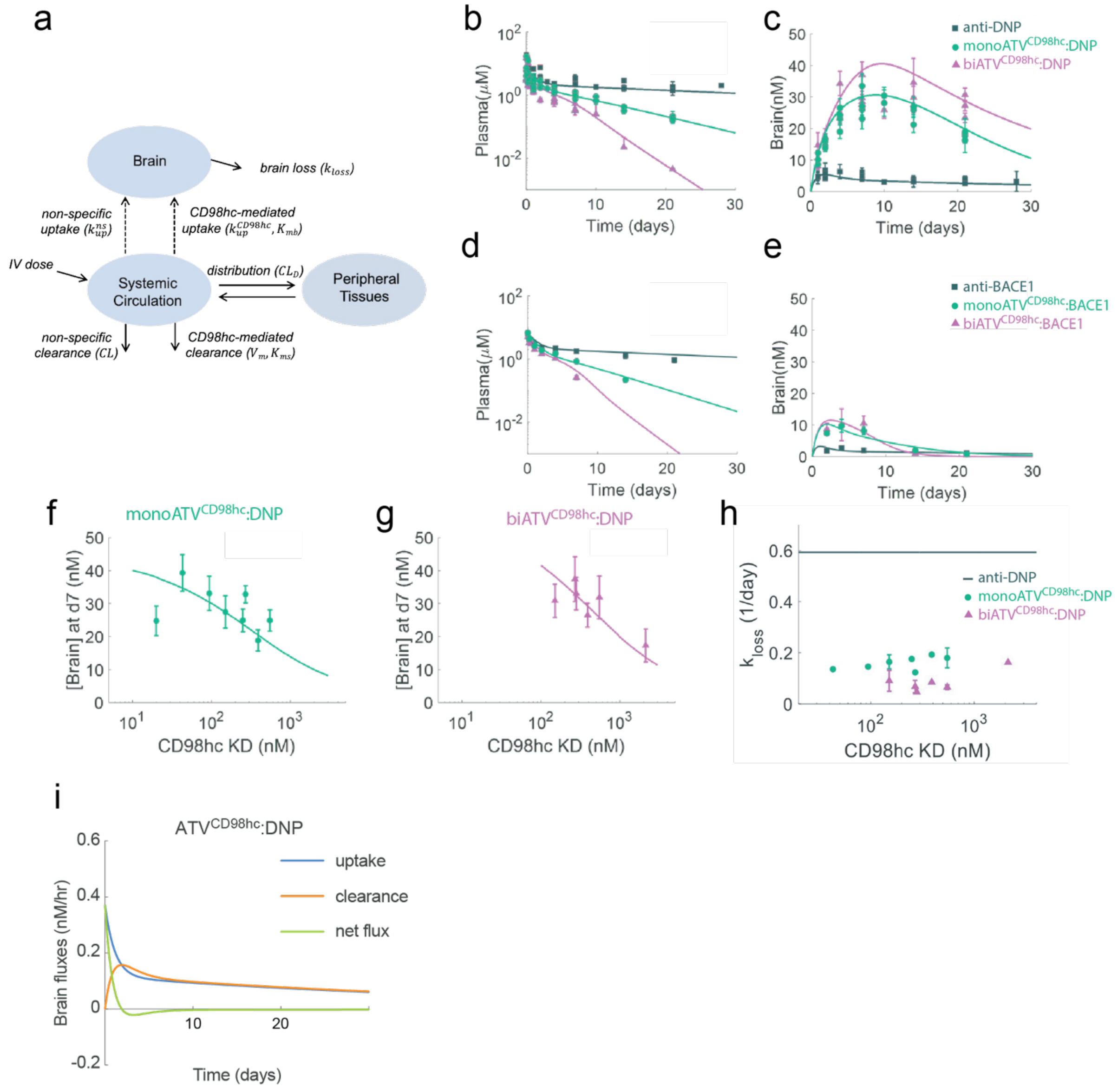

Supplemental Figure 13: Mathematical modeling of the systemic and brain PK of non-ATV and CD98hc TVs. a. Schematic of the PK model of non-ATV and ATV^{CD98hc}. **b-c.** Model fits (solid lines) to the observed huIgG concentrations of non-ATV as well as mono- and biATV^{CD98hc} in systemic circulation **(b,d)** and brain **(c,e)** across *in vivo* studies with ATV$^{CD98hc6.8}$:DNP (170 nM K_D) **(b,c)** and ATV$^{CD98hc6.39}$:BACE1 (94 nM K_D). Observed data is shown as mean ± SD, n=6 independent studies for anti-DNP and monoATV^{CD98hc}:DNP, n=3 independent studies for biATV^{CD98hc}:DNP. **(d,e)** Fabs at 50 mg/kg. The observed data in each study is represented as mean ± SD, n=5/group per timepoint. **f,g.** Model predictions (solid lines) overlaid on the observed concentrations of monovalent **(f)** and bivalent **(g)** ATV^{CD98hc}:DNP in the brain at day 7 post dose (50 mg/kg) as a function of CD98hc K_D. Observed data is shown as mean ± SD across *in vivo* studies, see Source Data file for n/group. **h.** Estimated rate constant for brain loss for mono- and biATV$^{CD98hc6.8}$:DNP shown as mean ± SD as a function of CD98hc K_D. Source data from f,g. **(i)** Simulated brain uptake, clearance, and total net flux in mouse for a 50 mg/kg IV dose of anti-DNP IgG.

Supplemental Table 3: Description of PK model parameters

Circulation PK		
**Parameter**	**Description**	**Units**
A_1	Amount in circulation	nmol/kg
A_2	Amount in peripheral tissues	nmol/kg
C_1	Concentration in circulation	μM
C_2	Concentration in peripheral tissues	μM
V_1	Circulating volume	mL/kg
V_2	Peripheral tissue volume	mL/kg
CL	Non-specific clearance	mL/day/kg
CL_D	Distribution clearance	mL/day/kg
V_m	Maximum CD98hc-mediated elimination rate	nmol/day/kg
K_{ms}	Michaelis Menten constant in circulation	μM
K_D	Affinity to CD98hc	μM
α	Calibration parameter	dimensionless
f_s	Factor for apparent affinity in circulation	dimensionless
Brain PK		
**Parameter**	**Description**	**Units**
C_b	Concentration in brain	μM
k_{up}^{ns}	Non-specific rate constant for brain uptake	1/day
k_{up}^{CD98hc}	Maximum CD98hc-mediated brain uptake rate	μM/day
K_{mb}	Michaelis Menten constant in brain	μM
f_b	Factor for apparent affinity in brain	dimensionless
k_{loss}	Rate constant for brain loss	1/day
$k_{loss,nonATV}$	Rate constant for brain loss for non-ATV molecules	1/day
$k_{loss,ATV}$	Rate constant for brain loss for ATV molecules	1/day

Supplemental Table 4: PK model parameter values

**Parameter**	**Units**	**Parameter estimate monoATV^{CD98hc}:DNP (CV%)**	**Parameter estimate biATV^{CD98hc}:DNP (CV%)**	**Parameter estimate monoATV^{CD98hc}:BACE1 (CV%)**	**Parameter estimate biATV^{CD98hc}:BACE1 (CV%)**
V_1	mL/kg	51.04 (2.55)	Same as monoATV^{CD98hc}:DNP	Same as monoATV^{CD98hc}:DNP	Same as biATV^{CD98hc}:DNP
V_2	mL/kg	97.86 (4.16)	Same as monoATV^{CD98hc}:DNP	Same as monoATV^{CD98hc}:DNP	Same as biATV^{CD98hc}:DNP
CL	mL/day/kg	2.66 (13.79)	Same as monoATV^{CD98hc}:DNP	Same as monoATV^{CD98hc}:DNP	Same as biATV^{CD98hc}:DNP
CL_D	mL/day/kg	27.22 (6.14)	81.09 (14.27)	Same as monoATV^{CD98hc}:DNP	Same as biATV^{CD98hc}:DNP
V_m	nmol/day/kg	34.54 (6.68)	Same as monoATV^{CD98hc}:DNP	Same as monoATV^{CD98hc}:DNP	Same as biATV^{CD98hc}:DNP
α	dimensionless	9.53 (11.5)	Same as monoATV^{CD98hc}:DNP	Same as monoATV^{CD98hc}:DNP	Same as biATV^{CD98hc}:DNP
f_s	dimensionless	1 (0)	0.22 (4.60)	Same as monoATV^{CD98hc}:DNP	Same as biATV^{CD98hc}:DNP
k_{up}^{ns}	1/day	0.001 (10.53)	Same as monoATV^{CD98hc}:DNP	Same as monoATV^{CD98hc}:DNP	Same as biATV^{CD98hc}:DNP
k_{up}^{CD98hc}	μM/day	0.0089 (5.65)	Same as monoATV^{CD98hc}:DNP	Same as monoATV^{CD98hc}:DNP	Same as biATV^{CD98hc}:DNP
f_b	dimensionless	1 (0)	0.66 (12.33)	Same as monoATV^{CD98hc}:DNP	Same as biATV^{CD98hc}:DNP
$k_{loss,nonATV}$	1/day	0.59 (12.54)	Same as monoATV^{CD98hc}:DNP	1.32 (7.90)	Same as monoATV^{CD98hc}:BACE1
$k_{loss,ATV}$	1/day	Supplemental Figure 11h	Supplemental Figure 11h	0.86 (7.80)	0.69 (7.16)

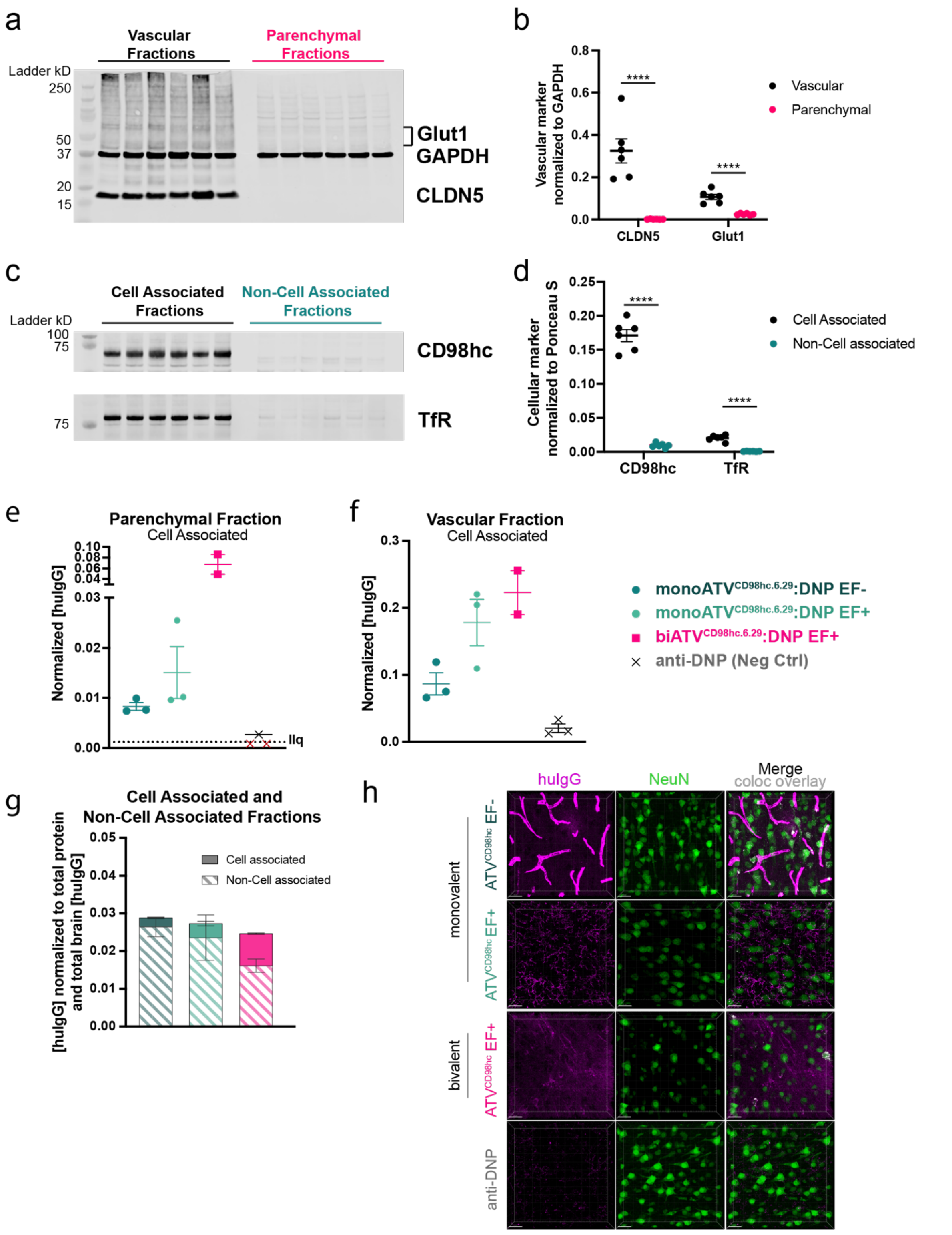

Supplemental Figure 14: Validation of capillary depletion fractions and biodistribution of ATV^{CD98hc} variants in cynomolgus monkey brain. a-b. Western blots and quantification demonstrating clean separation of the parenchymal fraction from vascular cells in cynomolgus monkey **c-d**. Western blots and quantification demonstrating depletion of cellular proteins from

the non-cell associated fraction in cynomolgus monkey **a-d** Two-tailed t-test ****= $p<0.0001$. See Supplemental Table 6 for exact p values. **e-f.** Concentrations of $ATV^{CD98hc6.29}$:DNP (205 nM K_D) variants in parenchymal and vascular fractions from cynomolgus brain. For huIgG concentration in the parenchymal fraction, 2 of 3 anti-DNP treated animals were below the lower limit of quantification. **g.** Concentrations of huIgG in cell associated and non-cell associated fractions. N=3 animals for $monoATV^{CD98hc}$:DNP groups, n=2 animals for $biATV^{CD98hc}$:DNP **a-f.** Graphs display mean ± SEM (for n=3); see Source Data file for n/group. **h.** Cynomolgus brain sections immunostained for huIgG (purple) and NeuN (green). Overlay is shown with colocalization pseudocolored white. Representative images are shown from n=3 animals/group (except for $biATV^{CD98hc.6.29}$:DNP where one animal was excluded), n=2 IHC sections/animal. Scale bars = 30 μm. Source data are provided as a Source Data file.

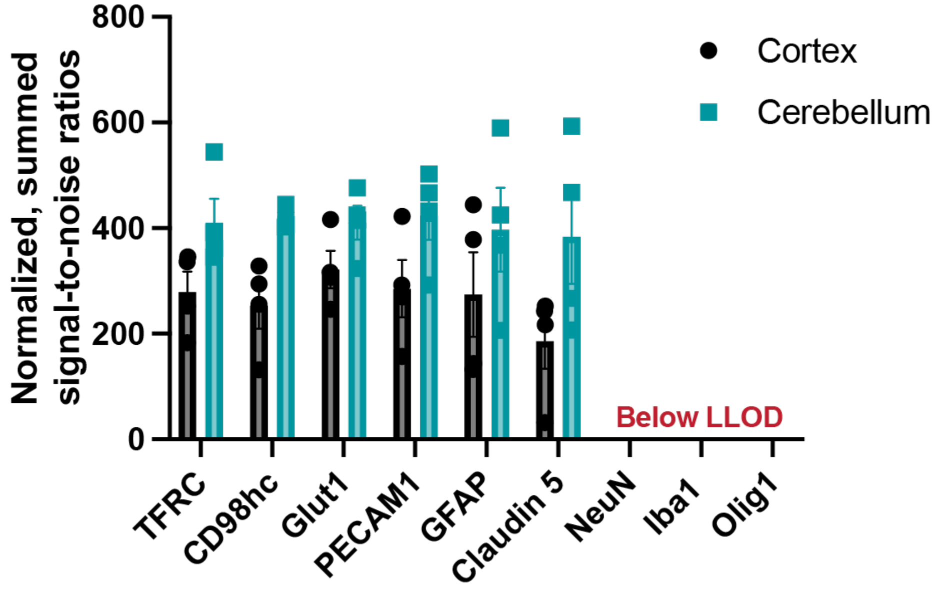

Supplemental Figure 15: Proteomics of isolated microvessel from human brain. Proteomics data from isolated human brain microvessels demonstrate high expression of known brain endothelial markers (Glut1, PECAM1, Claudin5) as well as RMT receptors TfR and CD98hc. Data represents n=4 (2M, 2F), mean ± SEM. Source data are provided as a Source Data file.

Supplemental Table 5: Overall statistical comparisons. For two-way ANOVAs, the test is performed on the interaction of group (denoted by color on the figures) and time point.

	ANOVAs				
**Figure and Panel**	**Type**	**Sample type**	**F**	**DF**	**P value**
Figure 3b	Two-way	Plasma	2.09	24	0.0042
Figure 3c	Two-way	Brain	14.22	16	4.65E-19
Figure 3d	Two-way	Parenchymal	5.99	8	1.29E-05
Figure 3e	Two-way	Vascular	5.80	8	2.05E-05
Figure 3f:	One way	Cell associated	4.593	3	0.0167
Figure 3f:	One way	Non-cell associated	25.47	3	2.5×10^{-6}
Figure 5a	Two-way	Plasma	0.62	14	0.8395
Figure 5b	Two-way	Brain	22.39	10	2.47E-18
Figure 5c	Two-way	Plasma	6.96	14	1.19E-09
Figure 5d	Two-way	Brain	10.40	8	5.30E-09
Figure 6a: C_{max}-C_{trough}	Two-way	Plasma	3.21	4	0.0236
Figure 6a: C_{trough}	Two-way	Plasma	43.56	4	2.55E-13
Figure 6b	One way	Brain	48	2	1.89E-06
Figure 6c: C_{max}-C_{trough}	Two-way	Plasma	1.81	4	0.1497
Figure 6c: C_{trough}	Two-way	Plasma	3.62	4	0.0146
Figure 6d	One way	Brain	190.0	2	7.99E-10
Figure 6e	One way	Monocytes	2.00	2	0.18
Figure 6f	One way	Lymphocytes	0.28	2	0.76
Figure 6g	One way	Reticulocytes	1.2	2	0.33
Figure 6h	One way	Monocytes	3.4	2	0.07
Figure 6i	One way	Lymphocytes	0.05	2	0.95
Figure 6j	One way	Reticulocytes	0.63	2	0.55
Supplemental Figure 11d	Two-way	Aβ40	11.39	8	1.18E-09
		**Unpaired T-Tests**			
		**Tailed**			**P value**
Supplemental 6b	CD31	2			4.2e-4
Supplemental 6b	CLDN5	2			6.0e-4
Supplemental 6d	CD98hc	2			1.1e-7
Supplemental 6d	TfR	2			7.2e-4
Supplemental 7q	1d	2			0.2172
Supplemental 7q	7d	2			0.9832
Supplemental 7q	21d	2			0.1246
Supplemental 7r	1d	2			0.2270

Supplemental 7r	7d	2	0.4343
Supplemental 7r	21d	2	0.4341
Supplemental 7s	1d	2	0.4144
Supplemental 7s	7d	2	0.5777
Supplemental 7s	21d	2	0.6209
Supplemental 7t	1d	2	0.2490
Supplemental 7t	7d	2	0.3542
Supplemental 7t	21d	2	0.5316
Supplemental 14b	CLDN5	2	2.1e-4
Supplemental 14b	Glut1	2	4.9e-5
Supplemental 14d	CD98hc	2	7.5e-9
Supplemental 14d	TfR	2	8.3e-7

Supplemental Table 6: Pairwise comparisons from Supplemental Table 5 ANOVAs. These comparisons are highlighted in the text.

	ANOVAs								
**Figure and Panel**	**Groups**	**Type**	**Units**	**estimate**	**df**	**conf.low**	**conf.high**	**T statistic**	**Adj P value**
Figure 3b	anti:DNP v biATV^{CD98hc}:DNP EF-	Two-way	µM	1.75	140	1.53	1.97	21.81	1.27E-14
Figure 3b	anti:DNP v biATV^{CD98hc}:DNP EF+	Two-way	µM	1.64	140	1.42	1.86	20.39	1.27E-14
Figure 3b	anti:DNP v monoATV^{CD98hc}:DNP EF-	Two-way	µM	1.31	140	1.08	1.53	16.26	1.27E-14
Figure 3b	anti:DNP v monoATV^{CD98hc}:DNP EF	Two-way	µM	1.10	140	0.88	1.32	13.72	1.27E-14
Figure 3b	biATV^{CD98hc}:DNP EF- v biATV^{CD98hc}:DNP EF+	Two-way	µM	-0.11	140	-0.34	0.11	-1.42	0.618
Figure 3b	biATV^{CD98hc}:DNP EF- v monoATV^{CD98hc}:DNP EF-	Two-way	µM	-0.45	140	-0.67	-0.22	-5.55	1.37E-06
Figure 3b	biATV^{CD98hc}:DNP EF+ v monoATV^{CD98hc}:DNP EF+	Two-way	µM	-0.54	140	-0.76	-0.31	-6.67	5.53E-09
Figure 3b	monoATV^{CD98hc}:DNP EF- v monoATV^{CD98hc}:DNP EF+	Two-way	µM	-0.20	140	-0.43	0.02	-2.53	0.089
Figure 3c	anti:DNP v biATV^{CD98hc}:DNP EF-	Two-way	nM	-18.84	99	-0.02	-0.02	-24.07	2.23E-10
Figure 3c	anti:DNP v biATV^{CD98hc}:DNP EF+	Two-way	nM	-25.12	99	-0.03	-0.02	-32.49	2.23E-10
Figure 3c	anti:DNP v monoATV^{CD98hc}:DNP EF-	Two-way	nM	-16.49	99	-0.02	-0.01	-21.33	2.23E-10
Figure 3c	anti:DNP v monoATV^{CD98hc}:DNP EF	Two-way	nM	-18.80	99	-0.02	-0.02	-24.31	2.23E-10
Figure 3c	biATV^{CD98hc}:DNP EF- v biATV^{CD98hc}:DNP EF+	Two-way	nM	-6.28	99	-0.01	0.00	-8.03	2.44E-10
Figure 3c	biATV^{CD98hc}:DNP EF- v monoATV^{CD98hc}:DNP EF-	Two-way	nM	2.35	99	0.00	0.00	3.00	0.028
Figure 3c	biATV^{CD98hc}:DNP EF+ v monoATV^{CD98hc}:DNP EF+	Two-way	nM	6.32	99	0.00	0.01	8.18	2.33E-10
Figure 3c	monoATV^{CD98hc}:DNP EF- v monoATV^{CD98hc}:DNP EF+	Two-way	nM	-2.30	99	0.00	0.00	-2.98	0.029
Figure 3d	anti:DNP v biATV^{CD98hc}:DNP EF-	Two-way	nM/total protein	-0.00013	59	-0.000181	-0.000087	-8.08	4.20E-10
Figure 3d	anti:DNP v biATV^{CD98hc}:DNP EF+	Two-way	nM/total protein	-0.00019	59	-0.000235	-0.000144	-11.66	1.80E-11
Figure 3d	anti:DNP v monoATV^{CD98hc}:DNP EF-	Two-way	nM/total protein	-0.00014	59	-0.000182	-0.000090	-8.37	1.46E-10
Figure 3d	anti:DNP v monoATV^{CD98hc}:DNP EF	Two-way	nM/total protein	-0.00015	59	-0.000199	-0.000108	-9.44	2.01E-11
Figure 3d	biATV^{CD98hc}:DNP EF- v biATV^{CD98hc}:DNP EF+	Two-way	nM/total protein	-0.00006	59	-0.000102	-0.000009	-3.34	0.012
Figure 3d	biATV^{CD98hc}:DNP EF- v monoATV^{CD98hc}:DNP EF-	Two-way	nM/total protein	-0.000002	59	-0.000049	0.000045	-0.13	1.000
Figure 3d	biATV^{CD98hc}:DNP EF+ v monoATV^{CD98hc}:DNP EF+	Two-way	nM/total protein	0.00004	59	-0.000010	0.000082	2.21	0.189
Figure 3d	monoATV^{CD98hc}:DNP EF- v monoATV^{CD98hc}:DNP EF+	Two-way	nM/total protein	-0.00002	59	-0.000063	0.000028	-1.07	0.821
Figure 3e	anti:DNP v biATV^{CD98hc}:DNP EF-	Two-way	nM/total protein	-0.00041	57	-0.000496	-0.000330	-13.97	1.12E-11
Figure 3e	anti:DNP v biATV^{CD98hc}:DNP EF+	Two-way	nM/total protein	-0.00048	57	-0.000563	-0.000397	-16.24	1.12E-11
Figure 3e	anti:DNP v monoATV^{CD98hc}:DNP EF-	Two-way	nM/total protein	-0.00025	57	-0.000330	-0.000166	-8.55	9.41E-11
Figure 3e	anti:DNP v monoATV^{CD98hc}:DNP EF	Two-way	nM/total protein	-0.00035	57	-0.000428	-0.000265	-11.95	1.12E-11
Figure 3e	biATV^{CD98hc}:DNP EF- v biATV^{CD98hc}:DNP EF+	Two-way	nM/total protein	-0.00007	57	-0.000150	0.000016	-2.26	0.172

Figure 3e	biATV^{CD98hc}:DNP EF- v monoATV^{CD98hc}:DNP EF-	Two-way	nM/total protein	0.00017	57	0.000083	0.000247	5.69	4.41E-06
Figure 3e	biATV^{CD98hc}:DNP EF+ v monoATV^{CD98hc}:DNP EF+	Two-way	nM/total protein	0.00013	57	0.000052	0.000215	4.61	2.21E-04
Figure 3e	monoATV^{CD98hc}:DNP EF- v monoATV^{CD98hc}:DNP EF+	Two-way	nM/total protein	-0.00010	57	-0.000179	-0.000019	-3.47	8.60E-03
Figure 3f: Cell associated	biATV^{CD98hc}:DNP EF- v monoATV^{CD98hc}:DNP EF-	One way	nM/total protein/brain [huIgG]	-0.004	16	-0.0071	3.314x 10^{-5}	2.778	0.0527
Figure 3f: Cell associated	biATV^{CD98hc}:DNP EF+ v monoATV^{CD98hc}:DNP EF+	One way	nM/total protein/brain [huIgG]	-0.003	16	-0.0067	0.0004	2.462	0.0984
Figure 3f: Cell associated	monoATV^{CD98hc}:DNP EF- v monoATV^{CD98hc}:DNP EF+	One way	nM/total protein/brain [huIgG]	-0.0002	16	-0.0038	0.0034	0.1653	0.9997
Figure 3f: Cell associated	biATV^{CD98hc}:DNP EF- v biATV^{CD98hc}:DNP EF+	One way	nM/total protein/brain [huIgG]	0.0002	16	-0.0034	0.0038	0.1505	0.9998
Figure 3f: Non-cell associated	biATV^{CD98hc}:DNP EF- v monoATV^{CD98hc}:DNP EF-	One way	nM/total protein/brain [huIgG]	0.0108	16	0.0064	0.0152	6.873	0.00001
Figure 3f: Non-cell associated	biATV^{CD98hc}:DNP EF+ v monoATV^{CD98hc}:DNP EF+	One way	nM/total protein/brain [huIgG]	0.0084	16	0.0040	0.0128	5.360	0.00026
Figure 3f: Non-cell associated	monoATV^{CD98hc}:DNP EF- v monoATV^{CD98hc}:DNP EF+	One way	nM/total protein/brain [huIgG]	-0.0019	16	-0.0025	0.0063	1.224	0.66399
Figure 3f: Non-cell associated	biATV^{CD98hc}:DNP EF- v biATV^{CD98hc}:DNP EF+	One way	nM/total protein/brain [huIgG]	0.0005	16	-0.0049	0.0039	0.2889	0.99750
Figure 5a	anti-DNP v monoATV^{CD98hc}:DNP	Two-way	µM	0.81	96	0.45	1.18	5.30	2.15E-06
Figure 5a	anti-DNP v monoATV^{TfR}:DNP	Two-way	µM	1.20	96	0.84	1.56	7.84	4.46E-10
Figure 5a	monoATV^{CD98hc}:DNP v monoATV^{TfR}:DNP	Two-way	µM	0.39	96	0.02	0.75	2.54	0.034
Figure 5b	anti-DNP v monoATV^{CD98hc}:DNP	Two-way	nM	-14.86	72	-16.88	-12.84	-17.61	<1.00E-10
Figure 5b	anti-DNP v monoATV^{TfR}:DNP	Two-way	nM	-5.05	72	-7.07	-3.03	-5.99	2.27E-07
Figure 5b	monoATV^{CD98hc}:DNP v monoATV^{TfR}:DNP	Two-way	nM	9.81	72	7.79	11.83	11.63	<1.00E-10
Figure 5c	anti-BACE v biATV^{CD98hc}:BACE1	Two-way	µM	1.09	96	0.93	1.24	16.84	4.28E-10
Figure 5c	anti-BACE v monoATV^{CD98hc}:BACE1	Two-way	µM	0.45	96	0.30	0.60	6.97	1.63E-09
Figure 5c	biATV^{CD98hc}:BACE1 v monoATV^{CD98hc}:BACE1	Two-way	µM	-0.64	96	-0.79	-0.48	-9.88	4.28E-10
Figure 5d	anti-BACE v biATV^{CD98hc}:BACE1	Two-way	nM	-4.27	60	-0.01	0.00	-9.14	2.17E-11
Figure 5d	anti-BACE v monoATV^{CD98hc}:BACE1	Two-way	nM	-3.78	60	0.00	0.00	-8.08	1.27E-10
Figure 5d	biATV^{CD98hc}:BACE1 v monoATV^{CD98hc}:BACE1	Two-way	nM	0.49	60	0.00	0.00	1.05	0.546
Figure 6a: Cmax-Ctrough	anti:DNP v monoATV^{CD98hc}:DNP EF-	Two-way	µM	-1.01	36	-1.72	-0.29	-3.45	0.004
Figure 6a: Cmax-Ctrough	anti:DNP v monoATV^{CD98hc}:DNP EF+	Two-way	µM	0.10	36	-0.61	0.81	0.34	0.939
Figure 6a: Cmax-Ctrough	monoATV^{CD98hc}:DNP EF- v monoATV^{CD98hc}:DNP EF+	Two-way	µM	1.11	36	0.39	1.82	3.79	0.002
Figure 6a: Ctrough	anti:DNP v monoATV^{CD98hc}:DNP EF-	Two-way	µM	2.61	36	2.31	2.91	21.19	<1.00E-10
Figure 6a: Ctrough	anti:DNP v monoATV^{CD98hc}:DNP EF+	Two-way	µM	2.44	36	2.13	2.74	19.74	<1.00E-10
Figure 6a: Ctrough	monoATV^{CD98hc}:DNP EF- v monoATV^{CD98hc}:DNP EF+	Two-way	µM	-0.18	36	-0.48	0.12	-1.44	0.331
Figure 6b:	anti:DNP v monoATV^{CD98hc}:DNP EF-	One-way	nM	-48.78	12	31.81	65.75	7.96	1.18E-05

Figure 6b:	anti:DNP v monoATV^{CD98hc}:DNP EF+	One-way	nM	-54.63	12	37.66	71.59	8.92	3.64E-06
Figure 6b:	monoATV^{CD98hc}:DNP EF- v monoATV^{CD98hc}:DNP EF+	One-way	nM	-5.85	12	-11.12	22.82	0.95	0.74
Figure 6c: Cmax-Ctrough	anti:DNP v biATV^{CD98hc}:DNP EF-	Two-way	µM	-2.49	34	-3.95	-1.03	-4.17	5.75E-04
Figure 6c: Cmax-Ctrough	anti:DNP v biATV^{CD98hc}:DNP EF+	Two-way	µM	-2.93	34	-4.43	-1.44	-4.81	8.68E-05
Figure 6c: Cmax-Ctrough	biATV^{CD98hc}:DNP EF- v biATV^{CD98hc}:DNP EF+	Two-way	µM	-0.44	34	-1.91	1.02	-0.74	0.739
Figure 6c: Ctrough	anti:DNP v biATV^{CD98hc}:DNP EF-	Two-way	µM	1.36	34	1.07	1.65	11.56	7.19E-13
Figure 6c: Ctrough	anti:DNP v biATV^{CD98hc}:DNP EF+	Two-way	µM	1.56	34	1.26	1.85	12.99	<1.00E-10
Figure 6c: Ctrough	biATV^{CD98hc}:DNP EF- v biATV^{CD98hc}:DNP EF+	Two-way	µM	0.20	34	-0.09	0.49	1.68	0.226
Figure 6d:	anti:DNP v biATV^{CD98hc}:DNP EF-	One-way	nM	-93.04	12	77.87	108.22	16.99	2.79E-09
Figure 6d:	anti:DNP v biATV^{CD98hc}:DNP EF+	One-way	nM	-92.34	12	77.16	107.51	16.86	3.04E-09
Figure 6d:	biATV^{CD98hc}:DNP EF- v biATV^{CD98hc}:DNP EF+	One-way	nM	0.71	12	-15.88	14.46	0.13	1.00
Supplemental Figure 11d	anti-BACE1 v biATV^{CD98hc}:BACE1	Two-way	pg/g	351.45	60	205.92	496.98	5.80	7.77E-07
Supplemental Figure 11d	anti-BACE1 v monoATV^{CD98hc}:BACE1	Two-way	pg/g	183.97	60	38.44	329.50	3.04	0.010
Supplemental Figure 11d	biATV^{CD98hc}:BACE1 v monoATV^{CD98hc}:BACE1	Two-way	pg/g	-167.48	60	-313.01	-21.94	-2.77	0.020

Supplemental Table 7: Antibodies used for western blot detection

Western Antibodies								
**Primary**				**Secondary**				
**Target**	**Company**	**Cat #**	**Dilution**	**Target**	**Company**	**Fluorophore**	**Cat #**	**Dilution**
mouse anti-CLDN5 (mouse and cynomolgus)	Invitrogen	35-2500	1:500	Goat anti-mouse	Li-Cor	800	926-32350	1:10000
Rabbit anti-CD31 (mouse)	Cell Signaling Technology	77699	1:1000	Donkey anti-rabbit	Li-Cor	680	926-68073	1:10000
Rabbit Glut1 (cynomolgus)	Novus	NB300-666	1:5000	Donkey anti-rabbit	Li-Cor	680	926-68073	1:10000
Rabbit anti-GAPDH	Abcam	Ab181603	1:15000	Donkey anti-rabbit	Li-Cor	680	926-68073	1:10000
Mouse anti-beta actin	Sigma	A2228-200UL	1:2000	Goat anti-mouse	Li-Cor	800	926-32350	1:10000
Mouse anti-TfR (mouse and cynomolgus)	Invitrogen	13-6800	1:2000	Goat anti-mouse	Li-Cor	800	926-32350	1:10000
Rabbit anti-CD98hc (human and cynomolgus)	Invitrogen	PA5-23661	1:1000	Goat anti-rabbit	Li-Cor	680	926-68023	1:10000
Rabbit anti-CD98hc (mouse)	LS Bio	LS-C296476	1:1000	Goat anti-rabbit	Li-Cor	680	926-68023	1:10000

Supplemental Table 8: Antibodies used for IHC staining

IHC Antibodies								
**Primary**				**Secondary**				
**Target**	**Company**	**Cat #**	**Dilution**	**Target**	**Company**	**Fluorophore**	**Cat #**	**Dilution**
				Human IgG (in mouse brain)	Jackson Immuno-research	647	109-605-003	1:500
				Human IgG (in cynomolgus brain)	Southern Biotech	647	2049-31	1:500
Iba1	Abcam	ab178846	1:500	Rabbit IgG	Invitrogen	488	A-11008	1:500
AQP4	Millipore	AB2218	1:500	Rabbit IgG	Invitrogen	488	A-11008	1:500
NeuN	Millipore	MAB377	1:500	Mouse IgG1	Invitrogen	488	A-21121	1:500
Glut1	Sigma	MABS132	1:500	Mouse IgG1	Invitrogen	568	A-21124	1:500
Mouse CD98hc (conjugated to Alex647)	Biolegend	128202	1:500					
Human/ cynomolgus CD98hc	Denali	-	1:1000	Human IgG	Jackson Immuno-research	488	109-545-003	1:500

Supplemental References:

1. F. Koentgen *et al.*, Exclusive transmission of the embryonic stem cell-derived genome through the mouse germline. *Genesis* **54**, 326-333 (2016).
2. S. van der Walt *et al.*, scikit-image: image processing in Python. *PeerJ* **2**, e453 (2014).
3. C. Sullivan, A. Kaszynski, PyVista: 3D plotting and mesh analysis through a streamlined interface for the Visualization Toolkit (VTK). *Journal of Open Source Software* **4**, (2019).
4. P. Virtanen *et al.*, SciPy 1.0: fundamental algorithms for scientific computing in Python. *Nat Methods* **17**, 261-272 (2020).
5. M. S. Kariolis *et al.*, Brain delivery of therapeutic proteins using an Fc fragment blood-brain barrier transport vehicle in mice and monkeys. *Sci Transl Med* **12**, (2020).
6. A. C. Boulay, B. Saubamea, X. Decleves, M. Cohen-Salmon, Purification of Mouse Brain Vessels. *J Vis Exp*, e53208 (2015).
7. W. Haas *et al.*, Optimization and use of peptide mass measurement accuracy in shotgun proteomics. *Mol Cell Proteomics* **5**, 1326-1337 (2006).
8. A. C. Tolonen, W. Haas, Quantitative proteomics using reductive dimethylation for stable isotope labeling. *J Vis Exp*, (2014).
9. G. C. McAlister *et al.*, MultiNotch MS3 enables accurate, sensitive, and multiplexed detection of differential expression across cancer cell line proteomes. *Anal Chem* **86**, 7150-7158 (2014).
10. J. D. Lapek, Jr., M. K. Lewinski, J. M. Wozniak, J. Guatelli, D. J. Gonzalez, Quantitative Temporal Viromics of an Inducible HIV-1 Model Yields Insight to Global Host Targets and Phospho-Dynamics Associated with Protein Vpr. *Mol Cell Proteomics* **16**, 1447-1461 (2017).
11. L. Ting, R. Rad, S. P. Gygi, W. Haas, MS3 eliminates ratio distortion in isobaric multiplexed quantitative proteomics. *Nat Methods* **8**, 937-940 (2011).
