# Supplementary material for: CD98hc is a target for brain delivery of biotherapeutics
Source: Nat Commun. 2023 Aug 19;14:5053. doi: 10.1038/s41467-023-40681-4 (PMC10439950; doi:10.1038/s41467-023-40681-4)
Supplement: Supplementary file 3 — Reporting Summary [file 41467_2023_40681_MOESM3_ESM.pdf]

nature portfolio

Corresponding author(s): Mihalis Kariolis, Joy Zuchero

Last updated by author(s): Jul 28, 2023

Reporting Summary

Nature Portfolio wishes to improve the reproducibility of the work that we publish. This form provides structure for consistency and transparency in reporting. For further information on Nature Portfolio policies, see our Editorial Policies and the Editorial Policy Checklist.

Statistics

For all statistical analyses, confirm that the following items are present in the figure legend, table legend, main text, or Methods section.

n/a	Confirmed	
☐	☒	The exact sample size (*n*) for each experimental group/condition, given as a discrete number and unit of measurement
☐	☒	A statement on whether measurements were taken from distinct samples or whether the same sample was measured repeatedly
☐	☒	The statistical test(s) used AND whether they are one- or two-sided<br>*Only common tests should be described solely by name; describe more complex techniques in the Methods section.*
☒	☐	A description of all covariates tested
☐	☒	A description of any assumptions or corrections, such as tests of normality and adjustment for multiple comparisons
☐	☒	A full description of the statistical parameters including central tendency (e.g. means) or other basic estimates (e.g. regression coefficient) AND variation (e.g. standard deviation) or associated estimates of uncertainty (e.g. confidence intervals)
☐	☒	For null hypothesis testing, the test statistic (e.g. *F*, *t*, *r*) with confidence intervals, effect sizes, degrees of freedom and *P* value noted<br>*Give P values as exact values whenever suitable.*
☒	☐	For Bayesian analysis, information on the choice of priors and Markov chain Monte Carlo settings
☒	☐	For hierarchical and complex designs, identification of the appropriate level for tests and full reporting of outcomes
☐	☒	Estimates of effect sizes (e.g. Cohen's *d*, Pearson's *r*), indicating how they were calculated

Our web collection on statistics for biologists contains articles on many of the points above.

Software and code

Policy information about availability of computer code

Data collection	Cell binding image collection: Harmony Software (PerkinElmer version 4.9), Western blot image collection: Image Studio (Li-Cor, Version 5.2.5), Peripheral tissue image acquisition: ZEN3.5 (Zeiss), Brain section image acquisition: Leica Application Suite X (3.5.7.23225)
Data analysis	SPR affinity measurments: Biacore Insight Evaluation software (version 2.0.15.12933), Cell binding analysis: Harmony Software (PerkinElmer version 4.9, Diffraction data processing: XDS (v20220110) and Phaser (v2.8.3), Crystal structure modeling: COOT (version 0.9.8.7) and in Refmac5 (v5.8.0403), Calculations of solvent accessible versus buried: PyMol (version 2.5.2), Pharmacokinetics analysis: Dotmatics software 5.5, Western blot analysis: Image Studio (Li-Cor, Version 5.2.5), brain section image processing: Imaris (v9.9.0, Bitplane), Mathematical Modeling: MATLAB 2022b, MathWorks, Generation of graphs Prism 9 (Graphpad, Version 9.5.1), Statistical analysis: Two-way ANOVAs: R version 4.2.0 with base packages and emmeans version 1.8.0, One-way ANOVAs and T-tests: Prism 9 (Graphpad, Version 9.5.1) for One-way, Proteomics data mining against UniProt: Sequest algorithm, Proteomics analysis: ProteomeDiscoverer (v2.1.0.81).

For manuscripts utilizing custom algorithms or software that are central to the research but not yet described in published literature, software must be made available to editors and reviewers. We strongly encourage code deposition in a community repository (e.g. GitHub). See the Nature Portfolio guidelines for submitting code & software for further information.

Data

Policy information about availability of data

All manuscripts must include a data availability statement. This statement should provide the following information, where applicable:

- Accession codes, unique identifiers, or web links for publicly available datasets
- A description of any restrictions on data availability
- For clinical datasets or third party data, please ensure that the statement adheres to our policy

The atomic coordinates for the TV6.6:CD98hc complex structure have been deposited in the PDB under the accession code 8G0M. Previously published crystal structures used in this study are available in the Protein Data Bank under accession codes 2DH2, 2DH3, 6IRS, 6IRT, 6JMQ, 6JMR, 6S8V, 7B00, 7DSK, 7DSL, and 4W4O. The mass spectrometry proteomics data have been deposited to the ProteomeXchange Consortium via the PRIDE partner repository with the dataset identifier PXD044159. Source Data are provided with this paper.

Human research participants

Policy information about studies involving human research participants and Sex and Gender in Research.

Reporting on sex and gender	N/A
Population characteristics	N/A
Recruitment	N/A
Ethics oversight	N/A

Note that full information on the approval of the study protocol must also be provided in the manuscript.

Field-specific reporting

Please select the one below that is the best fit for your research. If you are not sure, read the appropriate sections before making your selection.

☒ Life sciences ☐ Behavioural & social sciences ☐ Ecological, evolutionary & environmental sciences

For a reference copy of the document with all sections, see nature.com/documents/nr-reporting-summary-flat.pdf

Life sciences study design

All studies must disclose on these points even when the disclosure is negative.

Sample size	For in vivo studies, sample size was determined based on observed effect size. Each "n" represents animal number used for the study. For in vitro studies, sample size was determined based on observed effect size. Unless otherwise stated, each "n" is an independent experiment.
Data exclusions	Data was excluded if it was below the lower limit of a quantification or detection for a given assay and is so noted in figure legends. One animal was excluded from the study in cynomolgus monkeys due to signs of blood brain barrier disruption, which compromised the accuracy of measuring CD98hc-dependent brain uptake.
Replication	The exact number of repetitions (individual data points from each animal) are indicated in figures or figure legends.
Randomization	Animal studies were randomized by sex across groups. If age of animals varied more than 3 week, animals were also randomized by age across groups. Allocation for non-animal studies were randomized into experimental groups.
Blinding	The samples were not blinded during initial study planning to ensure that the number of groups of mice were randomized and balanced/ After initial assignments, animals were given number identifiers to blind investigators during downstream data collection and analysis. Mathematical modeling was conducted unblinded.

Reporting for specific materials, systems and methods

We require information from authors about some types of materials, experimental systems and methods used in many studies. Here, indicate whether each material, system or method listed is relevant to your study. If you are not sure if a list item applies to your research, read the appropriate section before selecting a response.

Materials & experimental systems

n/a	Involved in the study
☐	☒ Antibodies
☐	☒ Eukaryotic cell lines
☒	☐ Palaeontology and archaeology
☐	☒ Animals and other organisms
☒	☐ Clinical data
☒	☐ Dual use research of concern

Methods

n/a	Involved in the study
☒	☐ ChIP-seq
☒	☐ Flow cytometry
☒	☐ MRI-based neuroimaging

Antibodies

Antibodies used

human anti-human/cyno CD98hc (Denali Therapeutics, internally produced antibody)
Commercially available antibodies:
Invitrogen Chicken anti-cMyc, polyclonal (catalog #: A-21281)
Abcam rabbit anti-Iba1, clone: EPR16588 (catalog #: ab178846)
Millipore rabbit anti-AQP4, polyclonal (catalog #: AB2218)
Millipore Mouse IgG1 anti-NeuN, clone: A60 (catalog #: MAB377)
Sigma Mouse IgG1 anti-GLUT1, clone: 5B12.3 (catalog #: MABS132)
Biolegend rat anti-mouse CD98hc, clone: RL388 (catalog #: 128210)
Invitrogen Mouse anti-CLDN5, clone: 4C3C2 (catalog #: 35-2500)
Cell Signaling Technology Rabbit anti-CD31, clone: D8V9E (catalog #: 77699)
Novus Rabbit anti-Glut1, polyclonal (catalog #: NB300-666)
Abcam Rabbit anti-GAPDH, clone: EPR16884 (catalog #: Ab181603)
Invitrogen Mouse anti-TfR, clone: H68.4 (catalog #: 13-6800)
Invitrogen Rabbit anti-human CD98hc, polyclonal (catalog #: PA5-23661)
LS Bio Rabbit anti-mouse CD98hc, polyclonal (catalog #: LS-C296476)
Invitrogen Goat anti-chicken-488, polyclonal (catalog #: A-11039)
Jackson Immuno-research goat anti-human IgG-647, polyclonal (catalog #: 109-605-003)
Southern Biotech goat anti-human IgG-647, polyclonal (catalog #: 2049-31)
Invitrogen goat anti-rabbit-488, polyclonal (catalog #: A-11008)
Invitrogen goat anti-mouse IgG1-488, polyclonal (catalog #: A-21121)
Invitrogen goat anti-mouse IgG1-568, polylconal (catalog #: A-21124)
Li-Cor Goat anti-mouse-800 (catalog #: 926-32350)
Li-Cor Donkey anti-rabbit-680 (catalog #: 926-68073)
Li-Cor Goat anti-rabbit-680 (catalog #: 926-68023)

Validation

Invitrogen Chicken anti-cMyc (A-21281) is validated for flow cytometry based on 22 publications on manufacturer's website
Abcam Rabbit anti-Iba1 EPR16588 (ab178846) is validated based on 161 citations on manufacturer's website and in our hands based on expected staining pattern (Figure 4b, 7d, and Supplemental Figure 9b, f)
Millipore rabbit anti-AQP4 (AB2218) is validated based on 8 citations on manufacturer's websiteand in our hands based on expected staining pattern (Figure 4a, 7c, and Supplemental Figure 9a, c, and e)
Millipore Mouse IgG1 anti-NeuN A60 (MAB377) is validated based on 4471 citations on manufacturer's website and in our hands based on expected staining pattern (Supplemental Figure 10 and 14h)
Sigma Mouse IgG1 anti-GLUT1 5B12.3 (MABS132) is validated based on 3 citations on manufacturer's website and in our hands based on expected staining pattern (Supplemental Figure 5b and 9c)
Invitrogen Mouse anti-CLDN5 (35-2500) is validated for western blot based on 72 citations on the manufacturer's website and in our hands based on the expect band size (Supplemental Figures 6a and 14a)
Cell Signaling Technology Rabbit anti-CD31 (77699) is validated based on 196 citations on manufacturer's website and in our hands based on the expect band size (Supplemental Figure 6a)
Novus Rabbit anti-Glut1 (NB300-666) is validated for western blot based on 2 citations on the manufacturer's website and in our hands based on the expect band size (Supplemental Figure 14a)
Abcam Rabbit anti-GAPDH (Ab181603) is validated based on 146 citations on manufacturer's website and in our hands based on the expect band size (Supplemental Figure 6a, 14a)
Invitrogen Mouse anti-TfR (13-6800) is validated based on 497 citations on manufacturer's website and in our hands based on the expect band size (Supplemental Figure 6c, 14c)
Invitrogen Rabbit anti-human CD98hc (PA5-23661) is knockout validated in Supplemental Figure 5i, and is validated based on western blot band size in Supplemental Figures 6c and 14c
LS Bio Rabbit anti-mouse CD98hc (LS-C296476) is knockout validated in Supplemental Figure 5i
Biolegend rat anti-mouse CD98hc (128210) is validated based on 7 citations on manufacturer's website and by knockout in Supplemental Figure 5b-e
human anti human/cyno CD98hc (Denali Therapeutics) is knockout validated in Supplemental Figure 5b-e

Eukaryotic cell lines

Policy information about cell lines and Sex and Gender in Research

Cell line source(s)

HEK293 and HeLa were obtained from ATCC. CHO parent cells were obtained from ChemPartner (item discontinued). CHO stably expressing cyno CD98hc were generated from the parental CHO line (ChemPartner, custom order)

Authentication	HEK293, HeLa and CHO cell lines were not authenticated. CHO:cyCD98hc were validated for cyCD98hc expression using ICC with anti-human/cyno CD98hc antibody.
Mycoplasma contamination	Cell lines tested negative for mycoplasma contamination
Commonly misidentified lines (See ICLAC register)	None

Animals and other research organisms

Policy information about studies involving animals; ARRIVE guidelines recommended for reporting animal research, and Sex and Gender in Research

Laboratory animals	Mice: wild type (C57BL/6J), CD98hc mu/hu knockin (C57BL/6J), and CD98hc mu/hu;TfR mu/hu double knockin (C57BL/6J). Animals used in studies in this manuscript ranged from 1.2 - 5.0 months of age. Cynomologus monkeys: 22 to 48 months. Housing information (temperature and humidity) are included in Methods.
Wild animals	No wild animals were used in this study.
Reporting on sex	Mixed sex mice were used for all studies and were evenly distributed across experimental groups<br>In cynomologus monkey study only females were used.
Field-collected samples	No field collected samples were used in the study.
Ethics oversight	Mice: All procedures in animals were performed in adherence to ethical regulations and protocols approved by Denali Therapeutic Institutional Animal Care and Use Committee. Cynomolgus monkeys: Housing set-up is as specified in the USDA Animal Welfare Act (Code of Federal Regulations, Title 9) and as described in the Guide for the Care and Use of Laboratory Animals.

Note that full information on the approval of the study protocol must also be provided in the manuscript.
